# Post-Translational Modifications in NETosis and NETs-Mediated Diseases

**DOI:** 10.3390/biom9080369

**Published:** 2019-08-14

**Authors:** Hussein J. Hamam, Nades Palaniyar

**Affiliations:** 1 Program in Translational Medicine, Peter Gilgan Centre for Research and Learning, The Hospital for Sick Children, Toronto, ON M5G 0A4, Canada; 2 Department of Laboratory Medicine and Pathobiology, Faculty of Medicine, University of Toronto, Toronto, ON M5S 1A8, Canada; 3 Institute of Medical Sciences, Faculty of Medicine, University of Toronto, Toronto, ON M5S 1A8, Canada

**Keywords:** neutrophils, post translational modification, epigenetics, neutrophil extracellular trap formation, histone acetylation, histone citrullination, histone methylation, histone deacetylase inhibitors, apoptosis

## Abstract

Neutrophils undergo a unique form of cell death that generates neutrophil extracellular traps (NETs) that may help to neutralize invading pathogens and restore homeostasis. However, uncontrolled NET formation (NETosis) can result in numerous diseases that adversely affect health. Recent studies further elucidate the mechanistic details of the different forms of NETosis and their common end structure, as NETs were constantly found to contain DNA, modified histones and cytotoxic enzymes. In fact, emerging evidence reveal that the post translational modifications (PTMs) of histones in neutrophils have a critical role in regulating neutrophil death. Histone citrullination is shown to promote a rapid form of NET formation independent of NADPH oxidase (NOX), which relies on calcium influx. Interestingly, few studies suggest an association between histone citrullination and other types of PTMs to control cell survival and death, such as histone methylation. Even more exciting is the finding that histone acetylation has a biphasic effect upon NETosis, where histone deacetylase (HDAC) inhibitors promote baseline, NOX-dependent and -independent NETosis. However, increasing levels of histone acetylation suppresses NETosis, and to switch neutrophil death to apoptosis. Interestingly, in the presence of NETosis-promoting stimuli, high levels of HDACis limit both NETosis and apoptosis, and promote neutrophil survival. Recent studies also reveal the importance of the PTMs of neutrophils in influencing numerous pathologies. Histone modifications in NETs can act as a double-edged sword, as they are capable of altering multiple types of neutrophil death, and influencing numerous NET-mediated diseases, such as acute lung injury (ALI), thrombosis, sepsis, systemic lupus erythematosus, and cancer progression. A clear understanding of the role of different PTMs in neutrophils would be important for an understanding of the molecular mechanisms of NETosis, and to appropriately treat NETs-mediated diseases.

## 1. Introduction

The multicellular structure of the human body depends heavily on the innate immune system in the event of microbial infection. As neutrophils constitute the major components of the innate immunity, they are the first to respond against a large range of pathogens, such as bacteria and fungi [1]. However, they can be problematic in circulation or in tissues, depending on the intensity of the neutrophil influx. In the recent past, much attention has been directed towards neutrophil extracellular traps (NETs) formation for their relevance in mediating tissue injury, cancer progression, and inflammatory and autoimmune diseases, such as systemic lupus erythematosus (SLE) [2,3]. To form NETs (NETosis), neutrophils undergo a programmed cell death that releases extracellular DNA coated with antimicrobial proteins that could help to trap and clear pathogens [4].

However, the presence of excess NETs in the tissues often contributes to many pathologies. Numerous investigations during the last decade reveal the molecular mechanism of NETosis, including the NADPH oxidase 2 (NOX)-dependent and -independent NETosis [5]. Also, emerging evidence show a role of post-translational modifications in regulating neutrophil functions and cytotoxicity. Interestingly, the role of histone acetylation (e.g., acetylated histone H4, AcH4) was recently discovered in the context of NETosis [6]. By using histone deacetylase inhibitors (HDACis) to promote AcH4 levels, a dose-dependent switch of neutrophil death from NETosis to apoptosis was reported [7]. In this review, we will discuss the crucial role of neutrophils and NETs during infection and inflammation. Attention will also be directed towards the latest findings of histone modifications in altering neutrophil death. Lastly, we will discuss the impact of histone modifications during NET-mediated diseases.

## 2. Neutrophils in Health and Disease

### 2.1. Neutrophils

Neutrophils are terminally differentiated innate immune cells that possess highly condensed multi-lobulated nuclei [8]. These phagocytes act as the first line of defense in the innate immunity, as they are present in large numbers in the circulation [9,10]. Neutrophils originate from the hematopoietic cords that are located at the venous sinuses of the bone marrow [11]. They are derived from hematopoietic stem cells (HSCs), and are regulated by the granulocyte colony stimulating factor and a set of factors (e.g., PU1 is well-known that heterochromatin is regulated by post-translational modification (PTM)). For example, histone acetylation can decondense the chromatin and initiate transcription [12]. On the other hand, the methylation of histone is site-specific where it can either promote or repress gene expression. Emerging evidence suggests a role of PTMs during the progression of cells from quiescent to terminally-differentiated cells. As neutrophils differentiate, chromatin undergoes a large-scale remodeling which results in converting most of the euchromatin into condensed heterochromatin [13]. As a result, most genes responsible for housekeeping functions become repressed [14]. However, this is not always the case. PTMs levels between HSCs progenitor CD34^+^ cells and differentiated neutrophils are different. Histone methylation (histone H3 lysine 9 (H3K9)) is extensively present in CD34^+^ cells, but decreases to minimal levels as the neutrophil matures [15]. Other histone acetylation (H4K16) was also found to be significantly induced in mature neutrophils [16]. Therefore, understanding the role of PTM in neutrophils is a must, as chromatin change during maturation can result in a major transition in genome functioning [17].

It was previously thought that neutrophils are generated at times of acute inflammation, but studies show that they are steadily produced in the bone marrow [11,18]. In fact, about 2 × 10^11^ neutrophils are generated daily in an adult human body, and infection can result in a 10-fold increase in the production of these cells (up to 10^12^ cells daily) [19]. Mature neutrophils can either be stored in reservoirs, or be released into circulation. The bone marrow is considered the largest reservoir of neutrophils, but they can also be located as marginalized pools of granulocytes in the spleen, liver and lungs. In circulation, mature neutrophils have a diameter of 7–10 μm, and are traditionally known to have a short-live span with a brief circulating half-life of 6–8 h in humans and mice [20,21]. However, these estimates are challenged, as a recent study shows that circulating neutrophils have a lifespan of 18 h and 5.4 days, in mice and humans, respectively [22]. In addition, the lifespan of neutrophils increases significantly when they are activated during inflammation [23,24]. For example, the authors observed that photo-activated neutrophils had a lifespan of 48 h when they enter injured tissue in mice [25]. Furthermore, interpersonal variability and demographic factors (e.g., ethnicity) were shown to alter the circulating neutrophil counts [26]. PTM also has an impact on neutrophil’s survival [27].

### 2.2. Antimicrobial Functions of Neutrophils

Neutrophils possess different strategies to fight pathogens, including phagocytosis and degranulation. As pathogens breach the epithelium, host-derived proinflammatory chemokines (e.g., IL8, granulocyte chemotactic protein 2, and complement component C5a/C3a) and/or bacteria-derived mediators (e.g., lipoteichoic acid or N-formyl peptides) stimulate and recruit neutrophils from the bloodstream to the site of infection [28]. Neutrophil migration is a multi-step process that starts with the interaction between adhesion molecules and receptors found on both neutrophils and endothelial cells, which allows the cells to roll along and then firmly adhere to the endothelium, to extravasate, and then start interstitial migration [29,30]. In fact, PTMs are suggested to impact a neutrophil’s migration, as neutrophils lacking histone deacetylase (HDAC) 11 were shown to have a higher migratory capacity, compared to normal cells [31].

Neutrophils perform phagocytosis not only to engulf and eliminate pathogens, but it is also important during tissue remodeling and clearance of apoptotic and necrotic cells [32]. It is a multi-step process by which actin polymerization and membrane remodeling result in the containment of the internalized large particles (≥0.5 µm in diameter) in a membrane-derived vesicle termed a phagosome. The latter then undergoes a maturation process in order to create a toxic environment for most pathogens. This occurs by the fusion of granules containing antimicrobial contents with the phagosome. Emerging evidence shows a role of PTM in regulating phagocytosis, as neutrophils lacking HDAC11, for example, were shown to exert a higher phagocytic capacity [31].

Mature neutrophils contain three types of granules: Primary (azurophilic), secondary (specific), and tertiary (gelatinase) granules [33]. The categorization is based on the content and the time of granule formation relative to the process of the neutrophil maturation stages. Primary granules are the first to be formed, and contain myeloperoxidase (MPO) and serine proteases (e.g., NE and cathepsins). The second type of granules lack MPO, but have the potential to induce extracellular complement activation [34]. They also contain collagenase, gelatinase and lysozyme that are also found in tertiary granules, and they also contain lactoferrin. The three types of granules are produced and accumulated by the end of mature neutrophils. At times of phagocytosis, increased intracellular calcium levels trigger the fusion of the phagosome with the granules to form a phagolysosome.

The pathogen uptake triggers the initiation of the oxidative burst in neutrophils which result in the rapid production and release of reactive oxygen species (ROS) [35]. NOX and MPO have important roles during this process. The former is located on both the plasma and phagosome membranes, the activation of which results in the coupling of electrons with an oxygen molecule to generate a superoxide anion. The latter is converted by superoxide dismutase to hydrogen peroxide (H_2_O_2_), and then to hypochlorous acid (HOCl) by MPO [28].

Activated neutrophils also undergo an immune response called degranulation, which results in the release of granules [36]. The components of these granules have different antimicrobial functions. MPO-derived HOCl is considered the most bactericidal oxidant produced by neutrophils [28]. Lactoferrin also possesses bactericidal functions, and can be found in bodily secretions (e.g., saliva, tears and milk) [37]. In addition, granules possess defensins which increase the permeability of the bacteria [38]. NE, along with other serine proteases (e.g., proteinase 3 and cathepsin G), can digest pathogens and break down the extracellular matrix [28,39]. Furthermore, collagenase and gelatinase are metalloproteinases that work together to break down collagen [40]. Degranulation can also work alongside with phagocytosis, as granules fuse with phagosomes to facilitate pathogen elimination.

Neutrophils also possess an indirect immune defense mechanism that results in inducing inflammation [33]. Pro-inflammatory cytokines (e.g., TNF-α, IL-1β) and chemokines (e.g., IL-8, MIP-1α) are released by neutrophils upon the stimulation of regulatory cytokines (e.g., IL-4, IL-8, IL-13) [41,42]. These cytokines can also be regulated by PTM, as HDAC11, for example, was shown to repress the expression of pro-inflammatory cytokines [31]. Interestingly, these factors were shown to trigger another immune function of neutrophils as a form of cell death, termed “NETosis”.

## 3. Neutrophil Death

### 3.1. Neutrophil Apoptosis

To maintain homeostasis, the large number of neutrophils that are produced daily must be tightly regulated; otherwise, the antimicrobial peptides they possess can have devastating consequences, as they have the potential to create lethal environments [43]. To achieve this, circulating neutrophils are destined to die in a relatively short period of time, in a programmed form of cell death, termed apoptosis.

There are multiple signaling pathways that result in neutrophil apoptosis. Spontaneous apoptosis, induced by the intrinsic pathway, occurs at the mitochondrial level, and is initiated by the release of cytochrome C and other apoptotic factors into the cytosol upon the disruption of the outer membrane of the mitochondria [44]. However, the extrinsic pathway of apoptosis occurs as a result of the ligation of the surface death receptors that bind to TRAIL, TNFα, or Fas ligand. Neutrophils can also die by apoptosis upon killing microbes through phagocytosis, which is termed as phagocytosis-induced cell death [43]. Regardless of which signaling pathway is activated, the apoptotic neutrophil undergoes well-known morphological changes that include: DNA fragmentation, chromatin condensation, plasma membrane blebbing, and cell body shrinkage [45]. Apoptotic neutrophils are then cleared by the tissue macrophages or the macrophages residing in the spleen, liver and bone marrow.

Apoptosis is a heavily regulated mechanism in neutrophils. Inhibitor of apoptosis protein (IAP) is a group of regulatory factors that have the potential to suppress caspase activity [46]. One of the most important IAPs in neutrophils is the X-linked IAP (XIAP) that directly binds and inhibits procaspase-9 and -3 [45,47]. These procaspases are known to initiate intrinsic and extrinsic apoptotic signals, and to execute the proteolysis, respectively. Another important group of regulators is the Bcl-2 family, where Bax, Bak and Bid are pro-apoptotic/anti-survival proteins, while Bcl-2, McL-1, A1, and Bcl-XL are anti-apoptotic/pro-survival proteins [48,49]. Their roles were previously examined in vivo, as both Bax- and Bak-deficient mice had increased neutrophil counts, while mice lacking Bcl-2 showed regular neutrophil apoptosis [50,51]. Interestingly, neutrophils possess more pro-apoptotic factors as they mature. During earlier stages, neutrophils express inhibitors of the caspase pathway, such as XIAP, cellular IAPs and surviving, but at a lower rate as they mature [52,53]. Also, immature neutrophils contain all the previously mentioned pro-survival proteins, but are downregulated as neutrophils differentiate [54,55,56]. In fact, mature neutrophils only contain two Bcl-2 pro-survival proteins, Mcl-1 and A1 [49]. Also, apoptosis can be regulated through PTM, as histone acetylation (H4K16), for example, was shown to be enriched at specific DNA repeats which generated 50 kb DNA fragments during the first stages of programmed cell death in neutrophils [16].

In addition, the ROS has an important role in neutrophil apoptosis. It is well-known that increasing intracellular levels of ROS result in inducing apoptosis, as studies have shown that the treating neutrophils with NOX-derived ROS inhibitor (e.g., catalase) result in prolonging the life-span of these neutrophils [57,58]. Also, phagocytosis-induced ROS production was shown to activate caspase-3 and -8 [59]. However, there is another type of neutrophil cell death, NETosis, which is also activated by the increased intracellular ROS levels.

### 3.2. Neutrophil Extracellular Trap Formation (NETosis)

In the past two decades, neutrophils were shown to possess a unique immune response in a form of programmed cell death that is different from apoptosis and necrosis. In 1996, Takei et al. were the first to observe that dying neutrophils release chromatin-containing contents when they are activated with phorbol myristate acetate (PMA) [60]. Brinkmann et al. (2004) successfully demonstrated the functional relevance of this novel death process by neutrophils.

This phenomenon is termed as NETosis, since treating neutrophils with IL-8, lipopolysaccharide (LPS) or PMA results in the release of fibrous structures that resemble fishnets, and are termed as “NETs” [61]. By using scanning electron microscopy (SEM), the authors were able to structurally describe the NETs as fragile and smooth fibers that have the potential to aggregate into a thick bundle of fibers of diameters up to 50 nm. The original study also reports that NETs are membrane-free structures, and do not carry various cytoplasmic proteins (e.g., actin, microtubules, annexin I, and CD63; a granular membrane protein).

These web-like structures are made of DNA, modified histones and cytotoxic peptides/proteins [9,61]. DNA forms the main constituent, as treating NETs with deoxyribonuclease (DNase) is sufficient to disintegrate them. By using immunofluorescence imaging, studies confirm the presence of histone proteins on the NETs, including the core histones (H2A, H2B, H3, H4), linker histone H1 and the H2A-H2B-DNA complex. Also, some of the granule proteins have important roles during NETosis, as proteins found in the primary (NE, cathepsin G, MPO) and secondary/tertiary granules (lactoferrin, gelatinase) were also found on the NETs.

As NETosis was slowly getting accepted, many skeptics questioned how NETosis could have gone unnoticed so long, despite the numerous studies. Von Köckritz-Blickwede et al. (2008, 2009) claim that using fetal bovine serum (FBS) as a supplement culture media is problematic in neutrophil studies, as it contains DNase, which results in NETs degradation [62,63]. At the same time, controversies as to whether NETosis is an active immune response or just a regulated form of necrosis (necroptosis) sets the novel concept into question [64,65]. Later studies reveal that NETosis is a distinct mechanism, as the nuclear decondensation is accompanied by the dissociation of the nuclear envelope [66]. In necrosis, however, the chromatin de-condenses, and the nuclear contents spill out into the cytoplasm, while the nuclear membrane remains intact. Studies also confirm that NET formation is distinct from apoptosis, as caspases remain inactivated during the process [67,68]. On the other hand, recent literature reveals new types of NETosis that challenge the conventional understanding, such as the vital NETosis and ApoNETosis [64,69].

### 3.3. NOX-Dependent NETosis

As neutrophils are stimulated by NETotic inducers, such as PMA or LPS, intracellular ROS levels rapidly increase [8,70]. This occurs through NOX, as its activation rapidly generates superoxide and H_2_O_2_ by catalyzing the electron transfer from NADPH to oxygen. Neutrophils possess a multidomain complex enzyme, NOX2, which subunits gp91*^phox^* and p21*^rac^* form the core component called flavocytochrome b_558_ [71]. However, NOX activity is dependent on the protein kinase C (PKC)-dependent activation/phosphorylation of p47*^phox^*, p67*^phox^* and p21*^rac^* subunits and their complex assembly with b_558_ [72]. Notice that PKC can be activated by NETotic agonists (e.g., PMA) [73].

NOX-derived ROS production creates an optimal environment for two factors that are critical for NETosis, NE and MPO. The former is a neutrophil-specific serine protease that contributes to the antimicrobial activity in phagosomes at an optimal pH level of 7.5–8.5 (slightly alkaline) [74,75]. MPO catalyzes the oxidation of H_2_O_2_, and has optimal activity at the pH level 4.6–6.0 (slightly acidic). Both NE and MPO are stored in the primary granules of naïve neutrophils in association with azurocidin, cathepsin G, eosinophil cationic protein, defensin, lysosome, and lactoferrin as a complex termed azurosome [76]. Although NE’s function in the phagosome is independent of MPO, previous studies demonstrate that both are required for NETs formation [76]. In fact, individuals who are deficient in MPO were shown to be susceptible to opportunistic infections [77]. Also, neutrophils deficient in MPO or NE fail to undergo NETosis when stimulated with PMA [74,76].

Previous studies have shown that the increase in intracellular pH levels stimulates the ROS production and promotes histone H4 cleavage [78,79]. After 60 min from neutrophil stimulation, HOCl disassembles the azurosome, releasing NE, but not MPO, into the cytoplasm [76]. By 120 min post-stimulation, NE degrades F-actin and translocates into the nucleus, where it breaks down histone H1, so it can reach the core histones [74]. At this moment, NE and MPO facilitate the mixing of euchromatin and heterochromatin, which results in chromatin decondensation and the loss of lobular structure of the nucleus. Interestingly, histone H4, but not histone H3, was shown to be degraded by NE. Although MPO nuclear localization is independent of NE activity, it was previously shown that MPO and NE synergize to promote histone decondensation [74,80]. This results in transcription initiation, and together with ROS, the nuclear envelope disassembles into vesicles which result in a merge of both the cytoplasm and nucleoplasm. As the chromatin de-condense in the cytoplasm, they bind to granular and cytoplasmic antimicrobial proteins, such as NE and MPO, before rupturing the cytoplasmic membrane and allowing the liberation of NETs.

### 3.4. Other Types of NETosis

Although the majority of the studies demonstrate that NETs form in NOX-dependent manner, recent studies have shown that NETosis can occur through other pathways. In fact, few studies show that a fraction of neutrophils undergo vital NETosis and remain viable as they release NETs through granules, leaving behind short-lived enucleated neutrophils, termed cytoplasts [81,82,83]. Interestingly, these unique cells continue to show an immune response, as they are still able to perform chemotaxis, phagocytose and to clear pathogens. Initially, these observations were made in vitro, and were thought to be impossible to find in vivo. However, Yipp et al. (2012) showed that exposing mouse neutrophils to intradermal bacterial infection induced NET formation [82]. What was unique this time is that the NETosing cells remain alive and retain their antimicrobial characteristics, despite being enucleated.

Recent studies show that NETosis can also occur in the absence of NOX activity as the treatment with the NOX inhibitor, diphenyleneiodonium (DPI), only inhibits PMA- and LPS-induced NETosis [8,9]. Rather, NOX-independent NETosis requires an influx of extracellular calcium through calcium ionophores, such as A32178 and ionomycin secreted by the Gram-positive bacteria *Streptomyces conglobatus* [8,9]. Calcium ionophores were also shown to mobilize intracellular calcium pools, mainly from the endoplasmic reticulum [84]. Although NOX-independent NETosis does not utilize NOX-derived ROS, recent studies demonstrate that calcium ionophores induce mitochondrial ROS (mROS) production [9]. In fact, calcium ionophore-induced NETosis was dependent upon the mROS production, as treating neutrophils with mitochondrial uncouplers, dinitrophenol and MitoTEMPO, inhibit NOX-independent NETosis, but not PMA- or LPS-induced NETosis. This study also demonstrates that NOX-independent NETosis requires a potassium influx, mainly through the activation of SK3 channels. Compared to NOX-dependent NETosis, low and moderate levels of ERK and Akt activation were reported in NOX-independent NETosis, respectively, whereas similar levels of p38 activation were found in both pathways [8,9]. These studies also suggest that ERK activation is critical for the NOX-dependent pathway, while Akt activation is essential for NOX-independent NETosis.

## 4. Histone Modification in Neutrophils

Attention has recently been directed towards the role of neutrophils in modulating tissue injury and repair. Slaba et al. (2015) show that suppression of neutrophil recruitment to the liver injury site results in a delayed wound-healing process [85]. Other studies also confirmed the importance of neutrophils in normal tissue repair processes, such as the clearance of debris, normal revascularization and the production of the extracellular matrix [86,87]. In addition, neutrophils have critical roles in ensnaring and killing microbial pathogens extracellularly by inducing the formation of complement complexes [10]. A recent study has shown that although NETs recruit immune cells to the site of inflammation, bacteria trapped by NETs are shown to remain viable for a prolonged period, as NETs significantly reduce the bactericidal ability of the complement [88]. Also, studies demonstrate that chronic granulomatous disease (CGD) patients, who are unable to produce ROS, suffer from life-threatening disease for their neutrophil’s inability to form NETs [67,89,90]. However, uncontrolled NETosis or the accumulation of NETs results in NET-mediated inflammatory and autoimmune diseases. Emerging evidence supports a role of PTM, including histone citrullination, methylation and acetylation, in mediating the neutrophil’s immune functions and NET-mediated diseases.

### 4.1. Histone Citrullination

As mentioned earlier, histone decondensation is required for NET formation and release [9]. During the past decade, attention has been directed towards the role of peptidylarginine deiminase (PAD) in mediating histone citrullination (or deimination) in neutrophils. To date, there are five known PADs in humans [91]. However, studies confirm that only the 4th member, peptidyl arginine deiminase, type IV (PADI4), has the potential to deiminate histones inside the nucleus of neutrophils, as it bares the classical nuclear localization signal. As calcium ionophores increase the cytoplasmic calcium level, PADI4 forms a complex with calcium and gets activated, which rapidly translocates into the nuclei [92,93]. PADI4 catalyzes the conversion of positively-charged arginine present on histone H3 into neutral citrulline [94,95]. This results in disrupting the ionic interactions (e.g., hydrogen bonding) in the chromatin, and causes the histones to decondense. As the chromatin changes to a relaxed state, gene expression starts to take place. In fact, a study published by our lab has shown that transcription, but not translation, is required for NETosis [8].

Initial reports examine the potential of known neutrophil stimuli to induce citrullinated histone H3 (CitH3). Neeli et al. (2008) show that treating neutrophils in vitro with TNF, lipoteichoic acid, LPS, f-MLP, or H_2_O_2_ resulted in a rapid histone H3 deimination, which was identified as a component of NETosis, but was absent during apoptosis [92]. A later study confirms that CitH3 results in chromatin decondensation and is a result of PADI4 activity, as the inhibition of PADI4 decreases the histone H3 deimination and NET formation [94]. In addition, CitH3-mediated NETosis is dependent on the cell species, as canine neutrophils were shown to be dependent upon CitH3 to induce LPS- and PMA-stimulated NETosis [96]. However, studies report that human neutrophils behave in a different manner, especially in histone citrullination.

The requirement of PADI4 for increased intracellular calcium levels suggests that CitH3 would contribute exclusively during NOX-independent NETosis. Neeli and Radic (2013) show that treating human neutrophils with calcium ionophore promotes histone deimination [97]. However, PMA-induced NETosis fails to sufficiently promote CitH3, whereas A23187- and ionomycin-induced NETosis is sole in regulating PADI4, as alkaline conditions promote intracellular calcium influx, mitochondrial ROS levels and PADI4 activity to citrullinate histone H3, and eventually NET formation [79]. Kenny et al. (2017) also shows that citrullination is not required for the NET formation [98]. Treating primary human neutrophils with three PADI4 inhibitors suppresses spontaneous NETosis; however, NOX-independent NETosis remains intact. On the other hand, Li et al. (2010) demonstrates the importance of histone citrullination by using PADI4-deficient mice, as neutrophils were unable to undergo NETosis [95]. Despite these controversies, it is clear that histone citrullination plays an important role in histone unfolding and promoting transcription initiation, as CitH3 is now considered to be the hallmark of calcium-mediated NOX-independent NETosis.

### 4.2. Histone Methylation

Histone methylation in neutrophils can either promote or repress transcription, depending on the site of methylation, which can occur on all basic residues: arginines, lysines, and histidines [99,100,101]. Methylation of histones can either be mono (me1), di (me2), or tri (me3) on the amine group of the lysine residues, while arginine methylation can be mono, symmetrically dimethylated, or asymmetrically demethylated on their guanidinyl group [101,102,103,104].

Although histone methylation on histidine is reported to be rare and not well understood, studies show that it can be monomethylated [103,105]. Attention was recently directed towards examining lysine histone methylation at the following sites: Histone H3 lysine 4 (H3K4), H3K9, H3K27, H3K36, H3K79 and H4K20, while the sites of the arginine methylation include: H3R2, H3R8, H3R17, H3R26 and H4R3. In addition, histone methylation was shown to be reversible, and that some methylation events need to be maintained over time while others do not, depending on the biological context [102].

Histone methylation is the addition of methyl groups donated from S-adenosyl methionine to histones, and it is catalyzed by the histone methyltransferase (HMT) enzyme [101]. HMTs are classified into 3 families: HMTs that methylate lysines belong to either the SET domain-containing proteins or the Dot1 like proteins, while members of the PRMT family have been shown to methylate arginine [103,104,105]. In addition, a recent study has shown that calmodulin-lysine *N*-methyltransferase, which is a non-SET domain-containing protein, can methylate calmodulin and potentially histones, as well. The removal of methyl groups from histone lysine tails is catalyzed by histone demethylase (HDM) that is grouped into two families: The amine oxidases and the Jumonji C (JmjC) domain-containing, which are iron-dependent dioxygenases [102,106,107]. Arginine HDM is poorly understood. For example, JMJD6 is one of the JmjC domain proteins that was shown to demethylate arginine [108]. However, it is not exclusively considered an HDM, as its main function was shown to be the hydroxylation of RNA splicing factors [109]. In addition, while histone methylation at arginine residues promote transcription, the effects of histone lysine methylation on gene expression are dependent upon the histone site and the degree of methylation [110]. For example, H3K4, K36 and K79 are generally linked to transcription activation, while H3K9, K27 and H4K20 inhibit gene expression. Also, H3K4me1 acts at the transcription enhancer level, while H3K4me3 is found at the gene promoters. Another example is H4K20, where its me1 is observed in the bodies of active genes, while me3 is reported to repress gene expression by inducing histone compaction.

To date, there is no literature that indicates whether histone methylation can alter NETosis. However, some studies indicate the involvement of histone methylation, since treating human neutrophils with PMA results in increased H3K27me3 levels present on the NETs [111]. Liu et al. (2012) showed that treating primary human neutrophils with H_2_O_2_ alters the PTM, as harvested NETs showed increased H4K20me1, me2 and me3 [112]. However, histone methylation levels differ between different cell species, as treating murine cell-line derived neutrophils with H_2_O_2_, LPS, ionomycin or PMA resulted in increased H4K20me3 levels, but lower H4K20me2. Also, human neutrophils show stable levels of H3K9, H3K27 and H3K36 methylation, but were altered when murine cell-line derived neutrophils were treated with NETotic agonists. Based on these studies, it might be possible that the induction or suppression of histone methylation can alter NET formation.

Recently, attention has been directed towards the interaction between DNA and PTM, as studies show that DNA methylation and histone methylation can be dependent upon one another [113]. For example, it is known that H3K8 methyltransferase SUVH4 has the potential to bind to methylated DNA; however, a mutation in its methyl-DNA binding domain is shown to suppress H3K9me2 levels [114]. Hashimshony et al. (2003) constructed genetically-programmed methylated and unmethylated murine cells, and shows that the presence of DNA methylation results in H3K9 methylation while also suppressing that of H3K4, resulting in increased transcription [115]. Another study also demonstrates that increased DNA methylation results in suppressing H3K4 methylation, which is associated with active transcription [116].

In addition, histone methylation is regulated by other PTM, such as histone citrullination. In fact, it was suggested that citrullination competes with methylation at arginine residues in histones H3 and H4, where Wang et al. (2004) showed that human PADI4 has the potential to regulate histone arginine methylation in HL-60 granulocytes by converting methyl-arginine to citrulline [117]. However, conflicting data have been reported, where treating PADI4-deficient mice with methylated arginine synthetic peptides fails to remove the convert methylated arginine into citrulline in vitro [118]. Later studies made it clear that PADI4 activity varies in vitro and in vivo settings where PAD enzymes were shown to be relatively inefficient in vitro, but are capable to convert methylated arginine to citrulline in vivo [117,118,119].

### 4.3. Histone Acetylation

Histone acetylation is the transfer of an acetyl group from acetyl-CoA to histone lysine residue via a nucleophilic addition/elimination reaction which is catalyzed by histone acetyltransferase (HAT), which is grouped into 2 classes: A-type and B-type HATs [12]. The latter is responsible for acetylating newly synthesized histones in the cytoplasm, enabling them to enter the nucleus. On the other hand, A-type HATs contain a highly conserved motif, acetyl-CoA binding site, and they directly impact the transcriptional state for their ability to acetylate the histones of the nuclear chromatin. This group is subdivided into 3 subclasses based on the structural homology in their primary sequences: The Gcn5-related N-acetylase family, the MYST family and the orphan class for their lack of consensus HAT domain [120]. In general, HATs can acetylate the core histones which deed results in their decondensation. The relaxed conformation allows transcription factors to bind to the enhancer and promoter regions to initiate transcription. When compared to citrullination, genome-wide histone acetylation is expected to have a more pronounced effect on gene expression and therefore, NETosis might occur. In fact, Hollands et al. (2016) attempted to study the effect of a HAT inhibitor, anacardic acid, on neutrophils which resulted in induced NET formation [121]. However, anacardic acid has off-target effects, and significantly induces intracellular ROS production, similar to PMA. The authors showed that anacardic acid has no inhibitory effect on SUMOylation. On the other hand, Pandey et al. (2011) demonstrated that SUMOylation protects cells from oxidative stress, where small ubiquitin-like modifier-1 negatively regulates NOX-derived ROS production [122]. In addition, anacardic acid has the potential to induce autophagy, enhance apoptosis, and exhibit direct antimicrobial activity against multiple bacterial species, such as *S. aureus* and *H. pylori* [26,27,28,29,30]. Hollands et al. experimentally verified that anacardic acid-mediated NETs are bactericidal against multiple bacterial strains, including *S. aureus*. However, our recent study has demonstrated that bacteria trapped by NETs remain viable, as NETs have limited bactericidal properties [31]. Therefore, the effects of histone acetylation cannot be directly determined by anacardic acid, and it appears that inhibitors without off-target effects are required to elucidate the role of histone acetylation on NETosis.

Histone deacetylase complex (HDAC) catalyzes the removal of acetyl groups from lysine residues, resulting in compacting the chromatin and inhibiting the transcription factors from reaching the enhancer and promotor regions [12]. Up to date, studies have reported 18 different HDACs, which are grouped into 4 classes [123]. Class I includes HDACs 1, 2, 3 and 8, while class II members are 4, 5, 6, 7, 9 and 10 [124]. Class IV only contains HDAC 11. Members of class I, II, and III HDACs contain a zinc metal ion that is required to stabilize the base-mediated amide hydrolysis process. However, class III members, which are also known as sirtuins, require NAD^+^ to catalyze histone deacetylation, and are still under development [125]. In addition, neutrophils have been reported to significantly highly express all HDACs at the mRNA level, except for HDAC 5, 8, and 11 which are expressed at low levels [126].

The regulation of histone acetylation is also achieved by other PTMs at the level of both the DNA and chromatin. Hashimshony et al. (2003) studied the role of DNA methylation in regulating histone modifications [115]. DNA methylation is shown to have the potential to suppress the histone H4 acetylation and methylation of H3K4, while promoting H3K9me in order to negatively alter gene expression. On the other hand, HDACs can also influence DNA and histone methylation. Xuncheng et al. (2012) showed that HDAC6 is able to interact with DNA methyltransferase (DNMT) in vitro and in vivo, and HDAC6-deficient cells resulted in increased histone methylation (H3K4me2 and H3K4me3) and histone H3 and H4 acetylation [127,128]. In addition, studies have shown that histone citrullination and acetylation interact with one another. For example, PADI4 has the potential to efficiently demethylate the histone acetyltransferasep300, which enhances its acetylation activity to stimulate gene transcription [129,130]. In contrast, PADI4 is also shown to be dependent upon HDAC1 activity, where its knockdown resulted in decreased PADI4 and histone H3 citrullination levels, and increased histone arginine methylation [131].

Currently, there are five classes of HDACis, based on their chemical structure: Hydroxamates, short chain fatty acids, cyclic tetrapeptides, aliphatic acid and benzamides [132,133]. They can either be class specific or pan-deacetylase (pan HDACi), meaning that they are able to inhibit most of the HDAC classes. HDACis possess a similar structure to histone lysine tails. However, they form more stable bonds with a Zn^2+^ metal ion of the HDAC binding domain through conjugation and resonance [134]. Currently, there are only 3 pan HDACis that are clinically approved: Vorinostat, Belinostat, and Panobinostat.

Vorinostat (SAHA; trade name: Zolinza) is a linear hydroxamate compound that has the potential to inhibit most HDACs. Despite being the first to be approved by the United States Food and Drug Administration (FDA) for the treatment of T-cell lymphoma (TCL) in 2006, SAHA treatments result in harsh adverse effects (e.g., severe hematological side effects) [135,136,137]. On the other hand, Belinostat (PXD-101; trade name: Beleodaq) was granted an orphan drug status in 2009 (US), 2012 (EU) and was granted accelerated approval by the FDA, based upon its response rate, for treating peripheral TCL in 2014 [138]. It is used as monotherapy and in combination with chemotherapy drugs. It can be administered orally or intravenously (IV), and is reported to be extensively metabolized in the liver. To date, the acetylation levels of histones H3 and H4 are the most directly and widely used biomarkers in studies of HDACi, and Belinostat is shown to induce these levels in peripheral blood mononuclear cells (PBMC) in vitro and in vivo [139,140]. In fact, pharmacodynamic clinical assessments show that histone H4 hyperacetylation can be sustained for 4–24 h in PBMCs in a dose-dependent manner [141]. Belinostat is used in a large range of concentrations depending on its application [135]. For example, in vitro experiments show that it has a potent anti-tumor effect at a sub- to low-micromolar half maximal inhibitory concentration (IC50) potency in colon and ovarian cancer cells (0.2–3.4 µM), and to preferentially inhibit HDACs in tumor cells [138,139,142,143,144,145,146]. However, the IC50 range varies depending on the cell type examined, as Belinostat was shown to be effective at the 1.0–10.0 µM IC50 range against a panel of human bladder cancer cell lines [147]. For clinical applications, Belinostat is used at a recommended dose of 1,000 mg/m^2^ IV once daily for five consecutive days, resulting in a total dose of 5,000 mg/m^2^ per cycle [148]. Studies also indicate that ~92.9–95.8% of Belinostat can be bound to protein, and the maximum plasma concentration of Belinostat ranges from 88.2–174.4 µmol/L [138]. Panobinostat (LBH589) is the pan HDACi that is the most recently approved by the FDA in 2015 for treating multiple myeloma, and is currently under development for solid and hematological treatments [149,150]. Similar to Belinostat, Panobinostat can be administered orally or by IV, and both are able to inhibit most of the 18 different HDACs present in neutrophils. Accumulation of acetylated histones is observed earlier than Belinostat, with the time to achieve maximum levels at 2 h, and was shown to be maintained for 6–24 h [151]. Panobinostat has an effective anti-tumor potency from sub- to mid-nanomolar IC50 (2–530 nM) [151]. For in vivo application, Panobinostat is usually administered orally at doses ranging from 10–20 mg once daily for three doses per week, and ~90% of the drug can be bound to human plasma protein regardless of its concentration [150,152,153].

**Figure 1:**
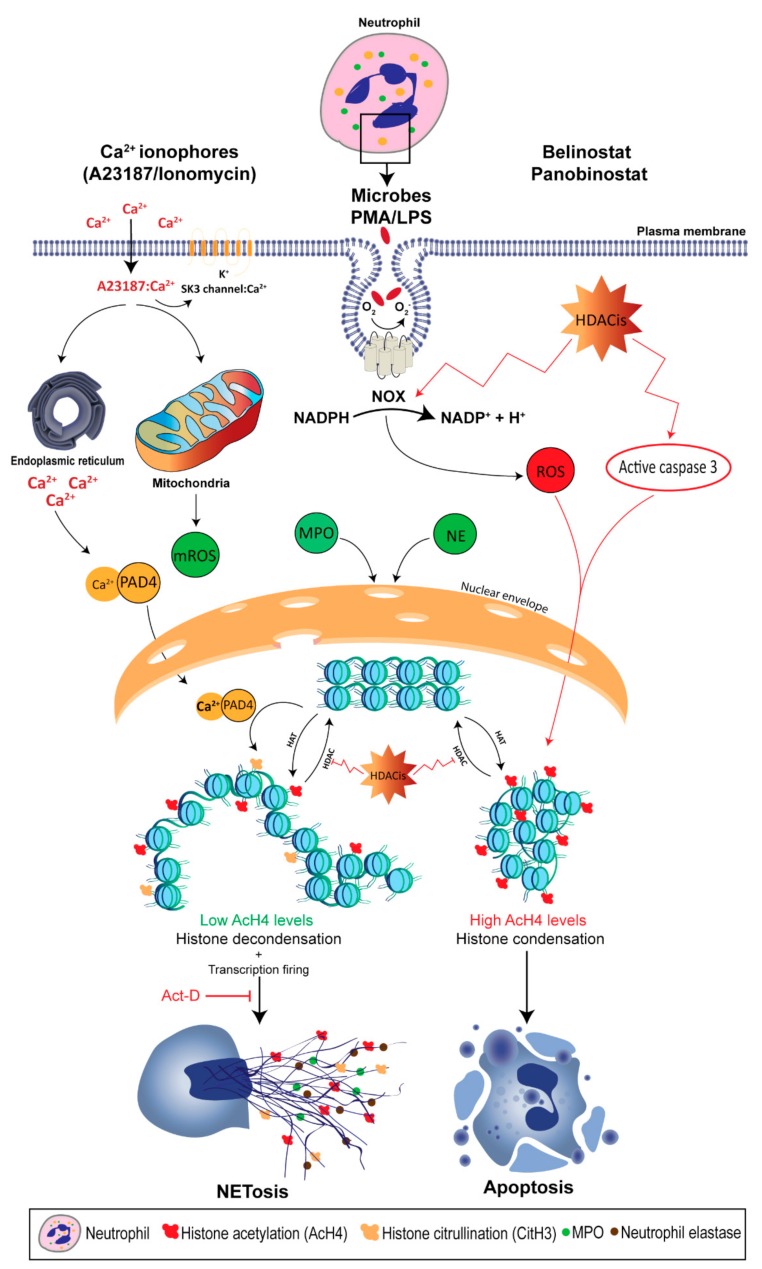
Citrullination (deimination) and acetylation in regulating neutrophil extracellular trap (NET) formation. Neutrophils can be activated during infection and inflammation to undergo NETosis. Calcium ionophores, such as A23187 and ionomycin, activate the NADPH oxidase (NOX)-independent, whereas phorbol myristate acetate (PMA) and lipopolysaccharide (LPS) induce NOX-dependent pathways of NETosis. Both pathways require increased levels of the reactive oxygen species (ROS) from NOX and/or mitochondrial origin, which results in kinase activation. After several intermediate steps, histones start to decondense and ultimately become NETs. During NOX-independent NETosis, increased intracellular Ca^2+^ influx activates peptidyl arginine deiminase, type IV (PADI4), which enables it to translocate into the nuclei. In the resting neutrophil nuclei, the negatively-charged DNA is tightly wrapped around the highly positively-charged histones (e.g., amino acids arginine and lysine) to form condensed chromatin. However, the active form of PADI4 (PADI4: Ca^2+^ complex) deiminate the positively-charged arginine into citrulline, which results in chromatin relaxation at promoters and transcription initiation. Similarly, histone deacetylase (HDAC) inhibitors (e.g., Belinostat and Panobinostat) are able to alter histone acetylation, where adding an acetyl group (H_3_C–C=O) to the tip of the side chain (–NH_3_) of the N-terminal lysines of histones (e.g., H4 histone with K5 acetylation; H4K5ac, or acetylated histone H4 (AcH4)), results in eliminating the positive charge (middle inset), weakening the overall chromatin structure, and enabling the entry of various proteins to access DNA (e.g., transcription machinery). By suppressing HDACs (18 in neutrophils), histone deacetylase inhibitors (HDACis) ultimately promote histone acetyl transferases (HAT) activity. This modification promotes baseline as well as NOX-dependent and -independent NETosis, without altering ROS production, as HDACis modify histones at a downstream level. However, increasing concentrations of HDACis induces NOX ROS production and activates the cleavage of caspase-3. As a result, neutrophils undergo apoptosis at a baseline condition. When the NETosis is induced with agonists, in the presence of high levels of AcH4, most of the neutrophils neither undergo NETosis nor apoptosis. In summary, histone citrullination and acetylation can alter NETosis and apoptosis, and the overall outcome depends upon the degree of the post-translational modification of histones, and the presence or absence of neutrophil death-inducing stimuli.

It was not until recently that scientists began to understand the direct effects of histone acetylation in regulating NET formation, as Belinostat and Panobinostat are used in a large range of concentrations depending on their application [135]. In fact, the clinical use of these HDACis can result in adverse effects; for example, a phase 2 study showed that 19 and 48% of patients diagnosed with myelodysplastic syndrome show grade 2 and 3–4 neutropenia when they were treated with Belinostat [154]. Therefore, elucidating the role of HDACis-mediated histone acetylation in altering neutrophil death is important. We have recently shown that treating primary human neutrophils with HDACis, either Belinostat or Panobinostat, results in dose-dependent histone hyperacetylation, and was observed in apoptotic and spontaneous, NOX-dependent and -independent NETotic cells [6,7]. This is consistent with the fact that AcH4 levels were increased upon treating in oligodendrocyte precursor cells and human cells (NB4 and HL-60) with SAHA and Belinostat [155,156]. Also, Belinostat and Panobinostat are shown to induce NETosis without altering intracellular ROS levels when they are used within their IC50 concentrations. However, higher levels (>1 μM Belinostat and >0.2 μM Panobinostat) of AcH4 result in a significant inhibition of all types of NETosis along with a dose-dependent increase in NOX-derived ROS levels. Interestingly, increasing concentrations of HDACis result in switching neutrophil death from NETosis to apoptosis in a dose-dependent manner (Figure 1). Studies have also shown that both Belinostat and Panobinostat induce apoptosis in HL60 cell lines in a dose-dependent manner [156,157]. However, results indicate that less than 30% of cells are apoptotic when cultured with Belinostat or Panobinostat for 24 h at concentrations within the IC50. On the other hand, these studies reported an increased number of cell deaths, with either higher concentrations of HDACi or with an increased duration of incubation (>24 h).

### 4.4. Role of Post-Translational Modifications in NET-Mediated Diseases

Emerging evidence suggests a role of uncontrolled NETosis in mediating chronic inflammation and several diseases, including acute lung injury (ALI), thrombosis, sepsis, autoimmune diseases (e.g., SLE) and cancer progression [33,76]. In fact, a significant body of emerging evidence supports a role for PTMs of these NET-mediated diseases. For example, neutrophils are key players to ALI, which is characterized by an increased microvascular permeability due to the disruption of alveolar-capillary morphology [158]. Interestingly, Liu et al. (2016) found that ALI is associated with increased CitH3 tissue levels in vivo, and DNase treatment significantly reduces CitH3 levels and degrades NETs [159]. As ALI progresses, lung tissues become damaged and scarred in a process termed “pulmonary fibrosis”. Emerging evidence shows that an HDACis (e.g., Trichostatin A, TSA) increases AcH4 and partially attenuates lung fibrosis [160]. In addition, it is well-known that NETosis is involved in thrombus formation, but emerging literature indicates that histone citrullination plays a key role [161]. In fact, Martinod et al. (2013) showed that CitH3 is crucial for pathological venous thrombosis in vivo, as only 10% of PADI4-deficient mice produced a thrombus, while 90% of wild-type mice did not [162].

### 4.5. Sepsis

Numerous studies indicate the relevance of NET formation in sepsis, which is defined as an overactive and toxic immune reaction that can result in fatal consequences, such as tissue damage and organ failure [163]. In fact, an emerging body of literature suggests that NET histones are the major contributors to endothelium injury and multiple organ dysfunction. Studies show that activated neutrophils have the potential to release NETs, which histones result in cytotoxic effects towards the epithelium in vitro and in vivo [164,165]. Saffarzadeh et al. (2012) confirmed the important role of histones in NETs, where DNA degradation in NET did not alter the NET-mediated cytotoxicity in sepsis [166].

There are many factors that regulate NET-mediated sepsis. For example, Mohammed et al. (2013) questioned whether vitamin C has a protective role in sepsis. By using vitamin C-deficient mice, the authors show that vitamin C suppresses PMA-induced NETosis and sepsis formation [167]. Interestingly, this study also showed that vitamin C protective potential is mediated through inhibition of PADI4-mediated CitH3. However, another study used PADI4-deficient mice and despite the fact that CitH3 levels were dependent upon PADI4 activity, NET-like structures were also seen in PADI4^−/−^ neutrophils [168]. The authors conclude against a role of PADI4 in the pathogenesis of sepsis. On the other hand, Biron et al. (2018) suggest that PADI4-mediated NETosis contributes to sepsis mortality, as PADI4^−/−^ mice result in decreased levels of proinflammatory mediators (e.g., IL-6 and TNF-α) and neutrophil influx into the lung [27]. In fact, it was recently shown that histone citrullination can be used as an early biomarker for detecting NETosis in sepsis-liver dysfunction [169].

Besides CitH3, attention has been directed towards the role of histone acetylation and methylation in sepsis. During normal conditions, HDAC and HAT activities are well-balanced. However, HDACs activity (e.g., HDAC6 and HDAC11) are off balanced during sepsis, which alters the transcription of pro- and anti-inflammatory genes (e.g., TNF-α and IL-10, respectively) in vitro and in vivo [31,170,171,172]. Interestingly, Wang et al. (2011) showed that treating PBMC with pan HDACis, such as Panobinostat, SAHA and TSA, results in a dose-dependent inhibition of IL-10 production, and relatively attenuates the immune paralysis during the hypo-inflammatory phase of sepsis [173,174]. Other studies demonstrate similar results, because the treatment of septic mice with HDACis, TSA and valproic acid, alleviates lung injury during sepsis [175,176]. In addition, H3K4 methylation is involved in the regulation of gene transcription that alters inflammation [177,178]. In fact, genome-wide mapping of HATs and DHACs show that methylation of H3K4 prime chromatin to undergo histone acetylation [179]. Studies have shown that inhibition of TREM protein (triggering receptor expressed on myeloid cells) by curcumin attenuates sepsis in vivo [180]. Interestingly, Yuan et al. (2012) demonstrated that curcumin-mediated sepsis alleviation is achieved by inhibiting H3K4 methylation and acetylation in TREM-1 promoter in vitro and in vivo [181].

### 4.6. Systemic Lupus Erythematosus (SLE)

SLE is an autoimmune disease which is characterized by chronic inflammation as a result of the immune system attacking the body’s own tissues and organs [182]. In fact, serum in normal conditions can degrade NETs; however, lupus patients possess autoantibodies that target DNA and histones, and this results in protecting NETs from being degraded by serum nucleases, which results in lupus nephritis [183]. The NET-DNA and -histone protection can be achieved by the complement deposition (e.g., C1q) and activation, which results in the suppression of DNase activity [184]. This results in increased exacerbations in SLE, ranging from rashes to seizures and psychosis.

Emerging evidence supports a role for histone citrullination in NET-mediated SLE pathology. Dwivedi et al. (2012) studied sera collected from SLE patients and tested for binding to NETs and found that increased levels of autoantibodies preferentially bound to citrullinated core histones in NETs, when compared to nondeiminated chromatin [185]. Interestingly, the same group also found that PADI4 has the potential to citrullinate linker histone H1, which was indicated in 6% of the sera of SLE patients [186]. Their results show that histone H1 citrullination results in the generation of new autoantibody epitopes that induce human B cells to produce autoantibodies. These claims were confirmed in vivo by using two mouse models of lupus, the MRL/lpr model and the New Zealand mixed model [187,188]. Both studies show that PADI4 inhibition with Cl-amidine results in a significant decrease of NETosis, protected against SLE pathologies (e.g., proteinuria) and improved endothelial cell differentiation and vasorelaxation.

In fact, studies have also shown a role of histone acetylation and methylation in SLE. Pieterse et al. (2014) analyzed the PTMs in NETs formed by SLE-derived neutrophils and found increased levels of histone acetylation and methylation, when compared to NETs from healthy donors [111]. Careful analysis reveals that acetylated levels of H4-K8, 12, 16 and H2B-K12 significantly increase as neutrophils derived from SLE patients are activated with PMA for 2 h. Interestingly, H3K27me3 levels show the greatest increase when compared to other PTMs in the same experimental conditions. However, these observations are exclusive to activated neutrophils which show an increased capacity to induce macrophages; however, histones found in unstimulated cells that were isolated from SLE patients show lower acetylated and methylated histones in NETs when compared to NETs from healthy donors. Similar results were found in another study which confirmed the role of histone PTM in NETs in inducing autoantibodies. Liu et al. (2012) profiled sera from patients with SLE and probed for 96 histones peptides with anti-human IgG antibodies [112]. By analyzing the composition of SLE-derived NETs, results showed that unmodified H2B, acetylated H2B-K12 and -K20 recorded the highest autoantibody binding reactivity. Interestingly, multiple methyl-histone H3 peptides and acetyl peptides of H2B, H3 and H4 also showed reactivity against IgM autoantibodies.

### 4.7. Cancer

It is well-known that HDACs control gene transcription, and therefore regulate cell proliferation, differentiation, G1 and G2/M cell cycle arrest, angiogenesis and apoptosis [133,189]. However, HDACs activity is not only limited by histone proteins, but can also act on non-histone proteins, such as molecular chaperones. Studies have shown that heat shock protein 90 (HSP90) is dysregulated by HDACs, altering the activity of many oncogenes, as well as p53, hypoxia-inducible factor 1-α and α-tubulin [190,191]. In fact, they are overexpressed in cancer cells, where HDACs regulate gene expression by silencing tumor suppressor genes [192]. In addition, class I HDACs are reported to regulate proliferation, as HDAC2 was shown to suppress apoptosis in tumor cells [193,194]. Therefore, HDAC inhibitors (HDACis) have recently emerged as an effective anticancer treatment.

Studies have shown that HDACis are able to disrupt the cell cycle, induce miRNA, inhibit angiogenesis, induce autophagy and induce apoptosis [192]. They are also able to overcome epigenetic resistance to chemotherapy drugs when used as a combination therapy. Beside cancerous cells, HDACis can also act on other cell types, namely neutrophils, which are present within the tumor microenvironments [195]. In fact, a growing number of studies report that NETosis induces cancer progression and metastasis [196]. Pieterse et al. (2017) showed that when endothelial cells have a limited capacity to internalize NETs, the persistent presence of NETs alter endothelial cell-cell contacts through the proteolysis of vascular endothelial-cadherin by neutrophil elastase [197]. This results in increased vascular leakage and transendothelial albumin passage. In addition, this study shows that NET-associated elastase induces a nuclear translocation of junctional β-catenin and promotes endothelial-to-mesenchymal transition. Another study by Cools-Lartigue et al. (2013) demonstrated that NETs are able to trap circulating lung carcinoma cells and induce the formation of hepatic micrometastases and gross metastatic disease [198]. In fact, these effects were abolished, either by degrading NETs with DNase, or by using neutrophil elastase inhibitor.

Attention has been directed towards the role of PTMs and cancer progression. Thalin et al. (2018) analyzed neutrophils isolated from patients with advanced cancer, which shows a significant increase in plasma CitH3, MPO and NE levels when compared with age-matched healthy individuals [199]. Interestingly, plasma CitH3 strongly predicted poor prognosis, and these elevations were exclusive to cancer progression, since they were not reported in non-cancerous, but in severely ill and hospitalized patients. As mentioned earlier, curcumin inhibits TREM-1 by suppressing the methylation and acetylation of H3K4, and studies have suggested that curcumin is also able to inhibit tumor growth [181,200]. In addition, Jiang et al. (2016) examined the clinical significance of histone methylation in esophageal cancer patients, and results show that increased levels of H3K9me2 are to be found in neutrophils when compared with samples acquired from trauma patients [201]. Another study examined the epigenetic alterations of the ALX/FPR_2_ gene, which activation exerts anti-inflammatory effects, and its potential in breast cancer treatment [202]. The authors showed that this gene is transcriptionally inaccessible in breast cancer cells, as they are characterized by low H3K27 acetylation and H3K4me3 levels, but high methylation at H3K27. However, the activation of p300 (HAT) and inhibition of DNMT result in chromatin decondensation and significantly induce ALX/FPR_2_ expression.

In addition, emerging evidence shows a potential pharmacological role of HDACis in cancer prevention and treatment. Wang et al. (2013) examined the effects of pan HDACis on pancreatic cancer cells, and found that treating cells with 10 μM Belinostat resulted in increased levels of ROS production and apoptotic cells [203]. Careful analysis reveals that Belinostat requires the activation of ROS-TAK1-AMPK signaling axis to mediate cancer-apoptosis effects. Similarly, Belinostat was shown to significantly, dose-dependently inhibit thyroid cancer cell proliferation through increased ROS production and the inhibition of RAS/RAF/ERK and PI3K/mTOR pathways [204]. Another study demonstrates that HDACis, such as Belinostat and Panobinostat, have the potential to significantly suppress leukemia and acute myelogenous leukemia cell growth [156,157].

## 5. Conclusions

Recent developments in neutrophil biology have shed light on the mechanisms that regulate cell death during healthy and disease conditions. The recent characterization of their antimicrobial functions demonstrates the critical role of NETs in the immune defense, where patients with compromised NET formation (e.g., CGD) are susceptible to fatal infections. In fact, emerging evidence shows a role of epigenetic regulation in controlling NETosis, especially histone citrullination and acetylation. However, the PTM of neutrophils can act as a double-edged sword, as they can alter multiple types of neutrophil cell death and influence numerous NET-mediated diseases (e.g., sepsis, SLE, and cancer progression). These recent findings can help improve our understanding of NET formation and be beneficial in designing therapies for NET-mediated diseases.

## Abbreviations

AcH4, acetylated histone H4; ALI, acute lung injuryCGD, granulomatous disease; CitH3, citrullinated histone H3; DNase, deoxyribonuclease; DNMT, DNA methyltransferase; DPI, diphenyleneiodonium; FDA, United States Food and Drug Administration; H_2_O_2,_ hydrogen peroxide; HAT, histone acetyltransferase; HDAC, histone deacetylase complex; HDACi, histone deacetylase inhibitor; HDM, histone demethylase; HMT, histone methyltransferase; HOCl, hypochlorous acid; HSCs, hematopoietic stem cells; H#K#, histone H# lysine #; H#R#, histone H# arginine #; IAPs, inhibitor of apoptosis protein; IC50, half maximal inhibitory concentration; iPS, induced pluripotent stem; IV, intravenously; JmjC, jumonji C; LBH589, panobinostat; LPS, lipopolysaccharide; MPO, myeloperoxidase; mROS, mitochondrial ROS; NE, neutrophil elastase; NETosis, NETs formation; NETs, neutrophil extracellular trap; NOX, NADPH oxidase 2; PAD, peptidylarginine deiminase; pan HDACi, pan-deacetylase inhibitors; PBMC, peripheral blood mononuclear cells; PKC, protein kinase C; PMA, phorbol myristate acetate; PTM, post-translational modification; PXD-101, belinostat; ROS, reactive oxygen species; SAHA, suberoylanilide hydroxamic acid; SLE, systemic lupus erythematosus; TAK1, transforming growth factor-β-activating kinase 1; TBP-2, thioredoxin-binding protein 2; TCL, T-cell lymphoma; TSA, trichostatin A; XIAP, X-linked IAP.

## References

[1] Mayadas T.N., Cullere X., Lowell C.A. (2014). The Multifaceted Functions of Neutrophils. Annu. Rev. Pathol. Mech. Dis..

[2] Downey D.G., Bell S.C., Elborn J.S. (2009). Neutrophils in cystic fibrosis. Thorax.

[3] Lee K.H., Kronbichler A., Park D.D.Y., Park Y.M., Moon H., Kim H., Choi J.H., Choi Y.S., Shim S., Lyu I.S. (2017). Neutrophil extracellular traps (NETs) in autoimmune diseases: A comprehensive review. Autoimmun. Rev..

[4] Yousefi S., Stojkov D., Germic N., Simon D., Wang X., Benarafa C., Simon H.U. (2019). Untangling “NETosis” from NETs. Eur. J. Immunol..

[5] Sollberger G., Tilley D.O., Zychlinsky A. (2018). Neutrophil Extracellular Traps: The Biology of Chromatin Externalization. Dev. Cell.

[6] Hamam H.J., Khan M.A., Palaniyar N. (2019). Histone Acetylation Promotes Neutrophil Extracellular Trap Formation. Biomolecules.

[7] Hamam H.J., Palaniyar N. (2019). Histone Deacetylase Inhibitors Dose-Dependently Switch Neutrophil Death from NETosis to Apoptosis. Biomolecules.

[8] Khan M.A., Palaniyar N. (2017). Transcriptional firing helps to drive NETosis. Sci. Rep..

[9] Douda D.N., Khan M.A., Grasemann H., Palaniyar N. (2015). SK3 channel and mitochondrial ROS mediate NADPH oxidase-independent NETosis induced by calcium influx. Proc. Natl. Acad. Sci. USA.

[10] Yuen J., Pluthero F.G., Douda D.N., Riedl M., Cherry A., Ulanova M., Kahr W.H.A., Palaniyar N., Licht C. (2016). NETosing neutrophils activate complement both on their own NETs and bacteria via alternative and non-alternative pathways. Front. Immunol..

[11] Liew P.X., Kubes P. (2019). The Neutrophil’s Role During Health and Disease. Physiol. Rev..

[12] Lu X., Wang L., Yu C., Yu D., Yu G. (2015). Histone Acetylation Modifiers in the Pathogenesis of Alzheimer’s Disease. Front. Cell. Neurosci..

[13] Grigoryev S.A., Bulynko Y.A., Popova E.Y. (2006). The end adjusts the means: Heterochromatin remodeling during terminal cell differentiation. Chromosom. Res..

[14] Kosak S.T., Groudine M. (2004). Form follows function: The genomic organization of cellular differentiation. Genes Dev..

[15] Lukášová E., Kořistek Z., Falk M., Kozubek S., Grigoryev S., Kozubek M., Ondřej V., Kroupová I. (2005). Methylation of histones in myeloid leukemias as a potential marker of granulocyte abnormalities. J. Leukoc. Biol..

[16] Urdinguio R.G., Lopez V., Bayón G.F., Diaz de la Guardia R., Sierra M.I., García-Toraño E., Perez R.F., García M.G., Carella A., Pruneda P.C. (2019). Chromatin regulation by Histone H4 acetylation at Lysine 16 during cell death and differentiation in the myeloid compartment. Nucleic Acids Res..

[17] Lukášová E., Kořistek Z., Klabusay M., Ondřej V., Grigoryev S., Bačíková A., Řezáčová M., Falk M., Vávrová J., Kohútová V. (2013). Granulocyte maturation determines ability to release chromatin NETs and loss of DNA damage response; these properties are absent in immature AML granulocytes. Biochim. Biophys. Acta Mol. Cell Res..

[18] Borregaard N. (2010). Neutrophils, from Marrow to Microbes. Immunity.

[19] Edwards S.W. (2010). The development and structure of mature neutrophils. Biochemistry and Physiology of the Neutrophil.

[20] Basu S., Hodgson G., Katz M., Dunn A.R. (2002). Evaluation of role of G-CSF in the production, survival, and release of neutrophils from bone marrow into circulation. Blood.

[21] Galli S.J., Borregaard N., Wynn T.A. (2011). Phenotypic and functional plasticity of cells of innate immunity: Macrophages, mast cells and neutrophils. Nat. Immunol..

[22] Pillay J., Den Braber I., Vrisekoop N., Kwast L.M., De Boer R.J., Borghans J.A.M., Tesselaar K., Koenderman L. (2010). In vivo labeling with ^2^H_2_O reveals a human neutrophil lifespan of 5.4 days. Blood.

[23] Summers C., Rankin S.M., Condliffe A.M., Singh N., Peters A.M., Chilvers E.R. (2010). Neutrophil kinetics in health and disease. Trends Immunol..

[24] Colotta F., Re F., Polentarutti N., Sozzani S., Mantovani A. (1992). Modulation of granulocyte survival and programmed cell death by cytokines and bacterial products. Blood.

[25] Wang J., Hossain M., Thanabalasuriar A., Gunzer M., Meininger C., Kubes P. (2017). Visualizing the function and fate of neutrophils in sterile injury and repair. Science.

[26] Hsieh M.M., Everhart J.E., Byrd-Holt D.D., Tisdale J.F., Rodgers G.P. (2007). Prevalence of neutropenia in the U.S. population: Age, sex, smoking status, and ethnic differences. Ann. Intern. Med..

[27] Biron B.M., Chung C.-S., Chen Y., Wilson Z., Fallon E.A., Reichner J.S., Ayala A. (2018). PADI4 Deficiency Leads to Decreased Organ Dysfunction and Improved Survival in a Dual Insult Model of Hemorrhagic Shock and Sepsis. J. Immunol..

[28] Teng T.-S., Ji A., Ji X.-Y., Li Y.-Z. (2017). Neutrophils and Immunity: From Bactericidal Action to Being Conquered. J. Immunol. Res..

[29] Rigby K.M., DeLeo F.R. (2012). Neutrophils in innate host defense against Staphylococcus aureus infections. Seminars in Immunopathology.

[30] Voisin M.B., Nourshargh S. (2013). Neutrophil transmigration: Emergence of an adhesive cascade within venular walls. J. Innate Immun..

[31] Sahakian E., Chen J., Powers J.J., Chen X., Maharaj K., Deng S.L., Achille A.N., Lienlaf M., Wang H.W., Cheng F. (2017). Essential role for histone deacetylase 11 (HDAC11) in neutrophil biology. J. Leukoc. Biol..

[32] Lee W.L., Harrison R.E., Grinstein S. (2003). Phagocytosis by neutrophils. Microbes Infect..

[33] Cheng O.Z., Palaniyar N. (2013). NET balancing: A problem in inflammatory lung diseases. Front. Immunol..

[34] Cowland J.B., Borregaard N. (2016). Granulopoiesis and granules of human neutrophils. Immunol. Rev..

[35] Roos D., Van Bruggen R., Meischl C. (2003). Oxidative killing of microbes by neutrophils. Microbes Infect..

[36] Sengeløv H., Follin P., Kjeldsen L., Lollike K., Dahlgren C., Borregaard N. (1995). Mobilization of granules and secretory vesicles during in vivo exudation of human neutrophils. J. Immunol..

[37] Masson P.L. (1969). Lactoferrin, an iron-binbing protein Ni neutrophilic leukocytes. J. Exp. Med..

[38] Rice W.G., Ganz T., Kinkade J.M., Selsted M.E., Lehrer R.I., Parmley R.T. (1987). Defensin-rich dense granules of human neutrophils. Blood.

[39] Campbell E.J., Campbell M.A., Owen C.A. (2000). Bioactive Proteinase 3 on the Cell Surface of Human Neutrophils: Quantification, Catalytic Activity, and Susceptibility to Inhibition. J. Immunol..

[40] Weiss S.J., Peppin G., Ortiz X., Ragsdale C., Test S.T. (1985). Oxidative autoactivation of latent collagenase by human neutrophils. Science.

[41] Kasama T., Miwa Y., Isozaki T., Odai T., Adachi M., Kunkel S. (2005). Neutrophil-Derived Cytokines: Potential Therapeutic Targets in Inflammation. Curr. Drug Targets Inflamm. Allergy.

[42] Kato T., Kitagawa S. (2006). Regulation of Neutrophil Functions by Proinflammatory Cytokines. Int. J. Hematol..

[43] McCracken J.M., Allen L.A.H. (2014). Regulation of human neutrophil apoptosis and lifespan in health and disease. J. Cell Death.

[44] Kennedy A.D., Deleo F.R. (2009). Neutrophil apoptosis and the resolution of infection. Immunol. Res..

[45] Taylor R.C., Cullen S.P., Martin S.J. (2008). Apoptosis: Controlled demolition at the cellular level. Nat. Rev. Mol. Cell Biol..

[46] Salvesen G.S., Duckett C.S. (2002). IAP proteins: Blocking the road to death’s door. Nat. Rev. Mol. Cell Biol..

[47] Squier M.K.T., Sehnert A.J., Sellins K.S., Malkinson A.M., Takano E., Cohen J.J. (1999). Calpain and calpastatin regulate neutrophil apoptosis. J. Cell. Physiol..

[48] Cory S., Adams J.M. (2002). The BCL2 family: Regulators of the cellular life-or-death switch. Nat. Rev. Cancer.

[49] Milot E., Filep J.G. (2011). Regulation of Neutrophil Survival/Apoptosis by Mcl-1. Sci. World J..

[50] Andina N., Conus S., Schneider E.M., Fey M.F., Simon H.U. (2009). Induction of Bim limits cytokine-mediated prolonged survival of neutrophils. Cell Death Differ..

[51] Villunger A., Scott C., Bouillet P., Strasser A. (2003). Essential role for the BH3-only protein Bim but redundant roles for Bax, Bcl-2, and Bcl-w in the control of granulocyte survival. Blood.

[52] Murphy B.M., O’Neill A.J., Adrain C., Watson R.W.G., Martin S.J. (2003). The Apoptosome Pathway to Caspase Activation in Primary Human Neutrophils Exhibits Dramatically Reduced Requirements for Cytochrome c. J. Exp. Med..

[53] Altznauer F., Martinelli S., Yousefi S., Thürig C., Schmid I., Conway E.M., Schöni M.H., Vogt P., Mueller C., Fey M.F. (2004). Inflammation-associated Cell Cycle–independent Block of Apoptosis by Survivin in Terminally Differentiated Neutrophils. J. Exp. Med..

[54] Weinmann P., Gaehtgens P., Walzog B. (1999). Bcl-Xl- and Bax-alpha-mediated regulation of apoptosis of human neutrophils via caspase-3. Blood.

[55] Santos-Beneit A.M., Mollinedo F. (2000). Expression of genes involved in initiation, regulation, and execution of apoptosis in human neutrophils and during neutrophil differentiation of HL-60 cells. J. Leukoc. Biol..

[56] Moulding D.A., Akgul C., Derouet M., White M.R., Edwards S.W. (2001). BCL-2 family expression in human neutrophils during delayed and accelerated apoptosis. J. Leukoc. Biol..

[57] Kasahara Y., Iwai K., Yachie A., Ohta K., Konno A., Seki H., Miyawaki T., Taniguchi N. (1997). Involvement of reactive oxygen intermediates in spontaneous and CD95 (Fas/APO-1)-mediated apoptosis of neutrophils. Blood.

[58] Aoshiba K., Yasui S., Hayashi M., Tamaoki J., Nagai A. (1999). Role of p38-mitogen-activated protein kinase in spontaneous apoptosis of human neutrophils. J. Immunol..

[59] Zhang B., Hirahashi J., Cullere X., Mayadas T.N. (2003). Elucidation of molecular events leading to neutrophil apoptosis following phagocytosis. Cross-talk between caspase 8, reactive oxygen species, and MAPK/ERK activation. J. Biol. Chem..

[60] Takei H., Araki A., Watanabe H., Ichinose A., Sendo F. (1996). Rapid killing of human neutrophils by the potent activator phorbol 12-myristate 13-acetate (PMA) accompanied by changes different from typical apoptosis or necrosis. J. Leukoc. Biol..

[61] Brinkmann V., Reichard U., Goosmann C., Fauler B., Uhlemann Y., Weiss D.S., Weinrauch Y., Zychlinsky A. (2004). Neutrophil Extracellular Traps Kill Bacteria. Science.

[62] Von Köckritz-Blickwede M., Goldmann O., Thulin P., Heinemann K., Norrby-Teglund A., Rohde M., Medina E. (2008). Phagocytosis-independent antimicrobial activity of mast cells by means of extracellular trap formation. Blood.

[63] Von Köckritz-Blickwede M., Chow O.A., Nizet V. (2009). Fetal calf serum contains heat-stable nucleases that degrade neutrophil extracellular traps. Blood.

[64] Yipp B.G., Kubes P. (2013). NETosis: How vital is it?. Blood.

[65] Berghe T.V., Linkermann A., Jouan-Lanhouet S., Walczak H., Vandenabeele P. (2014). Regulated necrosis: The expanding network of non-apoptotic cell death pathways. Nat. Rev. Mol. Cell Biol..

[66] Iba T., Hashiguchi N., Nagaoka I., Tabe Y., Murai M. (2013). Neutrophil cell death in response to infection and its relation to coagulation. J. Intensive Care.

[67] Fuchs T.A., Abed U., Goosmann C., Hurwitz R., Schulze I., Wahn V., Weinrauch Y., Brinkmann V., Zychlinsky A. (2007). Novel cell death program leads to neutrophil extracellular traps. J. Cell Biol..

[68] Remijsen Q., Berghe T.V., Wirawan E., Asselbergh B., Parthoens E., De Rycke R., Noppen S., Delforge M., Willems J., Vandenabeele P. (2011). Neutrophil extracellular trap cell death requires both autophagy and superoxide generation. Cell Res..

[69] Azzouz D., Palaniyar N. (2018). ApoNETosis: Discovery of a novel form of neutrophil death with concomitant apoptosis and NETosis. Cell Death Dis..

[70] Burg N.D., Pillinger M.H. (2001). The neutrophil: Function and regulation in innate and humoral immunity. Clin. Immunol..

[71] Rada B., Leto T. (2008). Oxidative innate immune defenses by Nox/Duox Family NADPH oxidases. Contrib. Microbiol..

[72] Brown D.I., Griendling K.K. (2009). Nox proteins in signal transduction. Free Radic. Biol. Med..

[73] Karlsson A., Nixon J.B., McPhail L.C. (2000). Phorbol myristate acetate induces neutrophil NADPH-oxidase activity by two separate signal transduction pathways: Dependent or independent of phosphatidylinositol 3-kinase. J. Leukoc. Biol..

[74] Papayannopoulos V., Metzler K.D., Hakkim A., Zychlinsky A. (2010). Neutrophil elastase and myeloperoxidase regulate the formation of neutrophil extracellular traps. J. Cell Biol..

[75] Khan M.A., Farahvash A., Douda D.N., Licht J.-C.C., Grasemann H., Sweezey N., Palaniyar N. (2017). JNK Activation Turns on LPS-And Gram-Negative Bacteria-Induced NADPH Oxidase-Dependent Suicidal NETosis. Sci. Rep..

[76] Metzler K.D., Goosmann C., Lubojemska A., Zychlinsky A., Papayannopoulos V. (2014). Myeloperoxidase-containing complex regulates neutrophil elastase release and actin dynamics during NETosis. Cell Rep..

[77] Nauseef W.M. (2007). How human neutrophils kill and degrade microbes: An integrated view. Immunol. Rev..

[78] Khan M.A., Philip L.M., Cheung G., Vadakepeedika S., Grasemann H., Sweezey N., Palaniyar N. (2018). Regulating NETosis: Increasing pH Promotes NADPH Oxidase-Dependent NETosis. Front. Med..

[79] De Souza C.N., Breda L.C.D., Khan M.A., de Almeida S.R., Câmara N.O.S., Sweezey N., Palaniyar N. (2018). Alkaline pH promotes NADPH oxidase-independent neutrophil extracellular trap formation: A matter of mitochondrial reactive oxygen species generation and citrullination and cleavage of histone. Front. Immunol..

[80] Vorobjeva N.V., Pinegin B.V. (2014). Neutrophil Extracellular Traps: Mechanisms of formation and role in health and disease. Biochemistry.

[81] Pilsczek F.H., Salina D., Poon K.K.H., Fahey C., Yipp B.G., Sibley C.D., Robbins S.M., Green F.H.Y., Surette M.G., Sugai M. (2010). A Novel Mechanism of Rapid Nuclear Neutrophil Extracellular Trap Formation in Response to Staphylococcus aureus. J. Immunol..

[82] Yipp B.G., Petri B., Salina D., Jenne C.N., Scott B.N.V., Zbytnuik L.D., Pittman K., Asaduzzaman M., Wu K., Meijndert H.C. (2012). Infection-induced NETosis is a dynamic process involving neutrophil multitasking in vivo. Nat. Med..

[83] Roos D., Voetman A.A., Meerhof L.J. (1983). Functional activity of enucleated human polymorphonuclear leukocytes. J. Cell Biol..

[84] Gupta A.K., Giaglis S., Hasler P., Hahn S. (2014). Efficient neutrophil extracellular trap induction requires mobilization of both intracellular and extracellular calcium pools and is modulated by cyclosporine A. PLoS ONE.

[85] Slaba I., Wang J., Kolaczkowska E., Mcdonald B., Lee W.Y., Kubes P. (2015). Imaging the dynamic platelet-neutrophil response in sterile liver injury and repair in mice. Hepatology.

[86] McDonald B., Kubes P. (2016). Innate Immune Cell Trafficking and Function During Sterile Inflammation of the Liver. Gastroenterology.

[87] Christoffersson G., Henriksnäs J., Johansson L., Rolny C., Ahlström H., Caballero-Corbalan J., Segersvärd R., Permert J., Korsgren O., Carlsson P.O. (2010). Clinical and experimental pancreatic islet transplantation to striated muscle: Establishment of a vascular system similar to that in native islets. Diabetes.

[88] Azzouz L., Cherry A., Riedl M., Khan M., Pluthero F.G., Kahr W.H.A., Palaniyar N., Licht C. (2018). Relative antibacterial functions of complement and NETs: NETs trap and complement effectively kills bacteria. Mol. Immunol..

[89] Brinkmann V., Zychlinsky A. (2012). Neutrophil extracellular traps: Is immunity the second function of chromatin?. J. Cell Biol..

[90] Bianchi M., Hakkim A., Brinkmann V., Siler U., Seger R.A., Zychlinsky A., Reichenbach J. (2009). Restoration of NET formation by gene therapy in CGD controls aspergillosis. Blood.

[91] Jones J.E., Causey C.P., Knuckley B., Slack-Noyes J.L., Thompson P.R. (2009). Protein arginine deiminase 4 (PADI4 ): Current understanding and future therapeutic potential. Curr. Opin. Drug Discov. Dev..

[92] Neeli I., Khan S.N., Radic M. (2014). Histone Deimination As a Response to Inflammatory Stimuli in Neutrophils. J. Immunol..

[93] Luo Y., Arita K., Bhatia M., Knuckley B., Lee Y.H., Stallcup M.R., Sato M., Thompson P.R. (2006). Inhibitors and inactivators of protein arginine deiminase 4: Functional and structural characterization. Biochemistry.

[94] Wang Y., Li M., Stadler S., Correll S., Li P., Wang D., Hayama R., Leonelli L., Han H., Grigoryev S.A. (2009). Histone hypercitrullination mediates chromatin decondensation and neutrophil extracellular trap formation. J. Cell Biol..

[95] Li P., Li M., Lindberg M.R., Kennett M.J., Xiong N., Wang Y. (2010). PADI4 is essential for antibacterial innate immunity mediated by neutrophil extracellular traps. J. Exp. Med..

[96] Li R.H.L., Ng G., Tablin F. (2017). Lipopolysaccharide-induced neutrophil extracellular trap formation in canine neutrophils is dependent on histone H3 citrullination by peptidylarginine deiminase. Vet. Immunol. Immunopathol..

[97] Neeli I., Radic M. (2013). Opposition between PKC isoforms regulates histone deimination and neutrophil extracellular chromatin release. Front. Immunol..

[98] Kenny E.F., Herzig A., Krüger R., Muth A., Mondal S., Thompson P.R., Brinkmann V., von Bernuth H., Zychlinsky A. (2017). Diverse stimuli engage different neutrophil extracellular trap pathways. eLife.

[99] Fischle W., Franz H., Jacobs S.A., Allis C.D., Khorasanizadeh S. (2008). Specificity of the chromodomain Y chromosome family of chromodomains for lysine-methylated ARK(S/T) motifs. J. Biol. Chem..

[100] Byvoet P., Shepherd G.R., Hardin J.M., Noland B.J. (1972). The distribution and turnover of labeled methyl groups in histone fractions of cultured mammalian cells. Arch. Biochem. Biophys..

[101] Murray K. (1963). The Occurrence of epsilon-N-Methyl Lysine in Histones. Biochemistry.

[102] Shi Y., Lan F., Matson C., Mulligan P., Whetstine J.R., Cole P.A., Casero R.A., Shi Y. (2004). Histone demethylation mediated by the nuclear amine oxidase homolog LSD1. Cell.

[103] Rea S., Eisenhaber F., O’Carroll D., Strahl B.D., Sun Z.W., Schmid M., Opravil S., Mechtier K., Ponting C.P., Allis C.D. (2000). Regulation of chromatin structure by site-specific histone H3 methyltransferases. Nature.

[104] Feng Q., Wang H., Ng H.H., Erdjument-Bromage H., Tempst P., Struhl K., Zhang Y. (2002). Methylation of H3-lysine 79 is mediated by a new family of HMTases without a SET domain. Curr. Biol..

[105] Bannister A.J., Kouzarides T. (2011). Regulation of chromatin by histone modifications. Cell Res..

[106] Cloos P.A.C., Christensen J., Agger K., Maiolica A., Rappsilber J., Antal T., Hansen K.H., Helin K. (2006). The putative oncogene GASC1 demethylates tri- and dimethylated lysine 9 on histone H3. Nature.

[107] Whetstine J.R., Nottke A., Lan F., Huarte M., Smolikov S., Chen Z., Spooner E., Li E., Zhang G., Colaiacovo M. (2006). Reversal of Histone Lysine Trimethylation by the JMJD2 Family of Histone Demethylases. Cell.

[108] Chang B., Chen Y., Zhao Y., Bruick R.K. (2007). JMJD6 is a histone arginine demethylase. Science.

[109] Webby C.J., Wolf A., Gromak N., Dreger M., Kramer H., Kessler B., Nielsen M.L., Schmitz C., Butler D.S., Yates J.R. (2009). Jmjd6 catalyses lysyl-hydroxylation of U2AF65, a protein associated with RNA splicing. Science.

[110] Greer E.L., Shi Y. (2012). Histone methylation: A dynamic mark in health, disease and inheritance. Nat. Rev. Genet..

[111] Pieterse E., Hofstra J., Berden J., Herrmann M., Dieker J., van der Vlag J. (2015). Acetylated histones contribute to the immunostimulatory potential of neutrophil extracellular traps in systemic lupus erythematosus. Clin. Exp. Immunol..

[112] Liu C.L., Tangsombatvisit S., Rosenberg J.M., Mandelbaum G., Gillespie E.C., Gozani O.P., Alizadeh A.A., Utz P.J. (2012). Specific post-translational histone modifications of neutrophil extracellular traps as immunogens and potential targets of lupus autoantibodies. Arthritis Res. Ther..

[113] Cedar H., Bergman Y. (2009). Linking DNA methylation and histone modification: Patterns and paradigms. Nat. Rev. Genet..

[114] Rajakumara E., Law J.A., Simanshu D.K., Voigt P., Johnson L.M., Reinberg D., Patel D.J., Jacobsen S.E. (2011). A dual flip-out mechanism for 5mC recognition by the Arabidopsis SUVH5 SRA domain and its impact on DNA methylation and H3K9 dimethylation in vivo. Genes Dev..

[115] Hashimshony T., Zhang J., Keshet I., Bustin M., Cedar H. (2003). The role of DNA methylation in setting up chromatin structure during development. Nat. Genet..

[116] Lande-Diner L., Zhang J., Ben-Porath I., Amariglio N., Keshet I., Hecht M., Azuara V., Fisher A.G., Rechavi G., Cedar H. (2007). Role of DNA methylation in stable gene repression. J. Biol. Chem..

[117] Wang Y., Wysocka J., Sayegh J., Lee Y.H., Pertin J.R., Leonelli L., Sonbuchner L.S., McDonald C.H., Cook R.G., Dou Y. (2004). Human PADI4 regulates histone arginine methylation levels via demethylimination. Science.

[118] Raijmakers R., Zendman A.J.W., Egberts W.V., Vossenaar E.R., Raats J., Soede-Huijbregts C., Rutjes F.P.J.T., van Veelen P.A., Drijfhout J.W., Pruijn G.J.M. (2007). Methylation of Arginine Residues Interferes with Citrullination by Peptidylarginine Deiminases in vitro. J. Mol. Biol..

[119] Cuthbert G.L., Daujat S., Snowden A.W., Erdjument-Bromage H., Hagiwara T., Yamada M., Schneider R., Gregory P.D., Tempst P., Bannister A.J. (2004). Histone deimination antagonizes arginine methylation. Cell.

[120] Roth S.Y., Denu J.M., Allis C.D. (2001). Histone acetyltransferases. Histone Acetyltransferases.

[121] Hollands A., Corriden R., Gysler G., Dahesh S., Olson J., Ali S.R., Kunkel M.T., Lin A.E., Forli S., Newton A.C. (2016). Natural product anacardic acid from cashew nut shells stimulates neutrophil extracellular trap production and bactericidal activity. J. Biol. Chem..

[122] Pandey D., Chen F., Patel A., Wang C.Y., Dimitropoulou C., Patel V.S., Rudic R.D., Stepp D.W., Fulton D.J. (2011). SUMO1 negatively regulates reactive oxygen species production from NADPH oxidases. Arterioscler. Thromb. Vasc. Biol..

[123] Johnstone R.W., Licht J.D. (2003). Histone deacetylase inhibitors in cancer therapy: Is transcription the primary target?. Cancer Cell.

[124] Yang X.-J., Grégoire S. (2005). Class II histone deacetylases: From sequence to function, regulation, and clinical implication. Mol. Cell. Biol..

[125] Chen L. (2011). Medicinal Chemistry of Sirtuin Inhibitors. Curr. Med. Chem..

[126] Kankaanranta H., Janka-Junttila M., Ilmarinen-Salo P., Ito K., Jalonen U., Ito M., Adcock I.M., Moilanen E., Zhang X. (2010). Histone deacetylase inhibitors induce apoptosis in human eosinophils and neutrophils. J. Inflamm..

[127] Liu X., Yu C.-W., Duan J., Luo M., Wang K., Tian G., Cui Y., Wu K. (2012). HDA6 Directly Interacts with DNA Methyltransferase MET1 and Maintains Transposable Element Silencing in Arabidopsis. Plant Physiol..

[128] Vaissière T., Sawan C., Herceg Z. (2008). Epigenetic interplay between histone modifications and DNA methylation in gene silencing. Mutat. Res. Rev. Mutat. Res..

[129] Lee Y.-H., Coonrod S.A., Kraus W.L., Jelinek M.A., Stallcup M.R. (2005). Regulation of coactivator complex assembly and function by protein arginine methylation and demethylimination. Proc. Natl. Acad. Sci. USA.

[130] Fuhrmann J., Thompson P.R. (2016). Protein Arginine Methylation and Citrullination in Epigenetic Regulation. ACS Chem. Biol..

[131] Denis H., Deplus R., Putmans P., Yamada M., Metivier R., Fuks F. (2009). Functional Connection between Deimination and Deacetylation of Histones. Mol. Cell. Biol..

[132] Dokmanovic M., Marks P.A. (2005). Prospects: Histone deacetylase inhibitors. J. Cell. Biochem..

[133] Marks P.A., Dokmanovic M. (2005). Histone deacetylase inhibitors: Discovery and development as anticancer agents. Expert Opin. Investig. Drugs.

[134] Lombardi P.M., Cole K.E., Dowling D.P., Christianson D.W. (2011). Structure, mechanism, and inhibition of histone deacetylases and related metalloenzymes. Curr. Opin. Struct. Biol..

[135] Yoon S., Eom G.H. (2016). HDAC and HDAC Inhibitor: From Cancer to Cardiovascular Diseases. Chonnam Med. J..

[136] Mann B.S., Johnson J.R., Cohen M.H., Justice R., Pazdur R. (2007). FDA Approval Summary: Vorinostat for Treatment of Advanced Primary Cutaneous T-Cell Lymphoma. Oncologist.

[137] Marks P.A., Xu W.S. (2009). Histone deacetylase inhibitors: Potential in cancer therapy. J. Cell. Biochem..

[138] Poole R.M. (2014). Belinostat: First global approval. Drugs.

[139] Molife L.R., de Bono J.S. (2011). Belinostat: Clinical applications in solid tumors and lymphoma. Expert Opin. Investig. Drugs.

[140] Plumb J.A., Finn P.W., Williams R.J., Bandara M.J., Romero M.R., Watkins C.J., La Thangue N.B., Brown R. (2003). Pharmacodynamic response and inhibition of growth of human tumor xenografts by the novel histone deacetylase inhibitor PXD101. Mol. Cancer Ther..

[141] Steele N.L., Plumb J.A., Vidal L., Tjørnelund J., Knoblauch P., Rasmussen A., Ooi C.E., Buhl-Jensen P., Brown R., Evans T.R.J. (2008). A phase 1 pharmacokinetic and pharmacodynamic study of the histone deacetylase inhibitor belinostat in patients with advanced solid tumors. Clin. Cancer Res..

[142] Tumber A., Collins L.S., Petersen K.D., Thougaard A., Christiansen S.J., Dejligbjerg M., Jensen P.B., Sehested M., Ritchie J.W.A. (2007). The histone deacetylase inhibitor PXD101 synergises with 5-fluorouracil to inhibit colon cancer cell growth in vitro and in vivo. Cancer Chemother. Pharmacol..

[143] Kong L.R., Tan T.Z., Ong W.R., Bi C., Huynh H., Lee S.C., Chng W.J., Eichhorn P.J.A., Goh B.C. (2017). Belinostat exerts antitumor cytotoxicity through the ubiquitin-proteasome pathway in lung squamous cell carcinoma. Mol. Oncol..

[144] Valiuliene G., Stirblyte I., Cicenaite D., Kaupinis A., Valius M., Navakauskiene R. (2015). Belinostat, a potent HDACi, exerts antileukaemic effect in human acute promyelocytic leukaemia cells via chromatin remodeling. J. Cell. Mol. Med..

[145] Qian X., Ara G., Mills E., LaRochelle W.J., Lichenstein H.S., Jeffers M. (2008). Activity of the histone deacetylase inhibitor belinostat (PXD101) in preclinical models of prostate cancer. Int. J. Cancer.

[146] Qian X. (2006). Activity of PXD101, a histone deacetylase inhibitor, in preclinical ovarian cancer studies. Mol. Cancer Ther..

[147] Buckley M.T., Yoon J., Yee H., Chiriboga L., Liebes L., Ara G., Qian X., Bajorin D.F., Sun T.-T., Wu X.-R. (2007). The histone deacetylase inhibitor belinostat (PXD101) suppresses bladder cancer cell growth in vitro and in vivo. J. Transl. Med..

[148] Lee H.Z., Kwitkowski V.E., Del Valle P.L., Ricci M.S., Saber H., Habtemariam B.A., Bullock J., Bloomquist E., Shen Y.L., Chen X.H. (2015). FDA approval: Belinostat for the treatment of patients with relapsed or refractory peripheral T-cell lymphoma. Clin. Cancer Res..

[149] (FDA), U.S.F. & D. administration Press Announcements - FDA approves Farydak for treatment of multiple myeloma. https://www.cancer.org/latest-news/fda-approves-farydak-panobinostat-for-multiple-myeloma.html.

[150] Garnock-Jones K.P. (2015). Panobinostat: First global approval. Drugs.

[151] Atadja P. (2009). Development of the pan-DAC inhibitor panobinostat (LBH589): Successes and challenges. Cancer Lett..

[152] Cheng T., Grasse L., Shah J., Chandra J. (2015). Panobinostat, a pan-histone deacetylase inhibitor: Rationale for and application to treatment of multiple myeloma. Drugs Today.

[153] Moore D. (2016). Panobinostat (Farydak): A Novel Option for the Treatment of Relapsed or Relapsed and Refractory Multiple Myeloma. Pharm. Ther..

[154] Cashen A., Juckett M., Jumonville A., Litzow M., Flynn P.J., Eckardt J., LaPlant B., Laumann K., Erlichman C., DiPersio J. (2012). Phase II study of the histone deacetylase inhibitor belinostat (PXD101) for the treatment of myelodysplastic syndrome (MDS). Ann. Hematol..

[155] Dincman T.A., Beare J.E., Ohri S.S., Gallo V., Hetman M., Whittemore S.R. (2016). Histone deacetylase inhibition is cytotoxic to oligodendrocyte precursor cells in vitro and in vivo. Int. J. Dev. Neurosci..

[156] Savickiene J., Treigyte G., Valiuliene G., Stirblyte I., Navakauskiene R. (2014). Epigenetic and molecular mechanisms underlying the antileukemic activity of the histone deacetylase inhibitor belinostat in human acute promyelocytic leukemia cells. Anticancer Drugs.

[157] Jiang X.J., Huang K.K., Yang M., Qiao L., Wang Q., Ye J.Y., Zhou H.S., Yi Z.S., Wu F.Q., Wang Z.X. (2012). Synergistic effect of panobinostat and bortezomib on chemoresistant acute myelogenous leukemia cells via AKT and NF-κB pathways. Cancer Lett..

[158] Matthay M.A., Ware L.B., Zimmerman G.A. (2012). The acute respiratory distress syndrome. J. Clin. Investig..

[159] Liu S., Su X., Pan P., Zhang L., Hu Y., Tan H., Wu D., Liu B., Li H., Li H. (2016). Neutrophil extracellular traps are indirectly triggered by lipopolysaccharide and contribute to acute lung injury. Sci. Rep..

[160] Ota C., Yamada M., Fujino N., Motohashi H., Tando Y., Takei Y., Suzuki T., Takahashi T., Kamata S., Makiguchi T. (2015). Histone deacetylase inhibitor restores surfactant protein-C expression in alveolar-epithelial type II cells and attenuates bleomycin-induced pulmonary fibrosis in vivo. Exp. Lung Res..

[161] Sperling C., Fischer M., Maitz M.F., Werner C. (2017). Neutrophil extracellular trap formation upon exposure of hydrophobic materials to human whole blood causes thrombogenic reactions. Biomater. Sci..

[162] Martinod K., Demers M., Fuchs T.A., Wong S.L., Brill A., Gallant M., Hu J., Wang Y., Wagner D.D. (2013). Neutrophil histone modification by peptidylarginine deiminase 4 is critical for deep vein thrombosis in mice. Proc. Natl. Acad. Sci. USA.

[163] Lipinska-Gediga M. (2017). Neutrophils, NETs, NETosis—Old or new factors in sepsis and septic shock?. Anestezjol. Intens. Ter..

[164] Xu J., Zhang X., Pelayo R., Monestier M., Ammollo C.T., Semeraro F., Taylor F.B., Esmon N.L., Lupu F., Esmon C.T. (2009). Extracellular histones are major mediators of death in sepsis. Nat. Med..

[165] Clark S.R., Ma A.C., Tavener S.A., McDonald B., Goodarzi Z., Kelly M.M., Patel K.D., Chakrabarti S., McAvoy E., Sinclair G.D. (2007). Platelet TLR4 activates neutrophil extracellular traps to ensnare bacteria in septic blood. Nat. Med..

[166] Saffarzadeh M., Juenemann C., Queisser M.A., Lochnit G., Barreto G., Galuska S.P., Lohmeyer J., Preissner K.T. (2012). Neutrophil extracellular traps directly induce epithelial and endothelial cell death: A predominant role of histones. PLoS ONE.

[167] Mohammed B.M., Fisher B.J., Kraskauskas D., Farkas D., Brophy D.F., Fowler A.A., Natarajan R. (2013). Vitamin C: A novel regulator of neutrophil extracellular trap formation. Nutrients.

[168] Claushuis T.A.M., van der Donk L.E.H., Luitse A.L., van Veen H.A., van der Wel N.N., van Vught L.A., Roelofs J.J.T.H., de Boer O.J., Lankelma J.M., Boon L. (2018). Role of Peptidylarginine Deiminase 4 in Neutrophil Extracellular Trap Formation and Host Defense during Klebsiella pneumoniae—Induced Pneumonia-Derived Sepsis. J. Immunol..

[169] Nomura K., Miyashita T., Yamamoto Y., Munesue S., Harashima A., Takayama H., Fushida S., Ohta T. (2019). Citrullinated Histone H3: Early Biomarker of Neutrophil Extracellular Traps in Septic Liver Damage. J. Surg. Res..

[170] Warford J., Lamport A.C., Kennedy B., Easton A.S. (2017). Human Brain Chemokine and Cytokine Expression in Sepsis: A Report of Three Cases. Can. J. Neurol. Sci..

[171] Alamdari N., Smith I.J., Aversa Z., Hasselgren P.-O. (2010). Sepsis and glucocorticoids upregulate p300 and downregulate HDAC6 expression and activity in skeletal muscle. Am. J. Physiol. Regul. Integr. Comp. Physiol..

[172] Cheng F., Lienlaf M., Perez-Villarroel P., Wang H.W., Lee C., Woan K., Woods D., Knox T., Bergman J., Pinilla-Ibarz J. (2014). Divergent roles of histone deacetylase 6 (HDAC6) and histone deacetylase 11 (HDAC11) on the transcriptional regulation of IL10 in antigen presenting cells. Mol. Immunol..

[173] von Knethen A., Brüne B. (2019). Histone Deacetylation Inhibitors as Therapy Concept in Sepsis. Int. J. Mol. Sci..

[174] Wang H., Cheng F., Woan K., Sahakian E., Merino O., Rock-Klotz J., Vicente-Suarez I., Pinilla-Ibarz J., Wright K.L., Seto E. (2011). Histone Deacetylase Inhibitor LAQ824 Augments Inflammatory Responses in Macrophages through Transcriptional Regulation of IL-10. J. Immunol..

[175] Zhang L., Jin S., Wang C., Jiang R., Wan J. (2010). Histone deacetylase inhibitors attenuate acute lung injury during cecal ligation and puncture-induced polymicrobial sepsis. World J. Surg..

[176] Ji M.H., Li G.M., Jia M., Zhu S.H., Gao D.P., Fan Y.X., Wu J., Yang J.J. (2013). Valproic acid attenuates lipopolysaccharide-induced acute lung injury in mice. Inflammation.

[177] Kouzarides T. (2007). Chromatin modifications and their function. Cell.

[178] Klose R.J., Zhang Y. (2007). Regulation of histone methylation by demethylimination and demethylation. Nat. Rev. Mol. Cell Biol..

[179] Wang Z., Zang C., Cui K., Schones D.E., Barski A., Peng W., Zhao K. (2009). Genome-wide mapping of HATs and HDACs reveals distinct functions in active and inactive genes. Cell.

[180] Gibot S., Alauzet C., Massin F., Sennoune N., Faure G.C., Béné M., Lozniewski A., Bollaert P., Lévy B. (2006). Modulation of the Triggering Receptor Expressed on Myeloid Cells—1 Pathway during Pneumonia in Rats. J. Infect. Dis..

[181] Yuan Z., Syed M.A., Panchal D., Rogers D., Joo M., Sadikot R.T. (2012). Curcumin mediated epigenetic modulation inhibits TREM-1 expression in response to lipopolysaccharide. Int. J. Biochem. Cell Biol..

[182] Rahman A., Isenberg D.A. (2008). Systemic Lupus Erythematosus. N. Engl. J. Med..

[183] Hakkim A., Furnrohr B.G., Amann K., Laube B., Abed U.A., Brinkmann V., Herrmann M., Voll R.E., Zychlinsky A. (2010). Impairment of neutrophil extracellular trap degradation is associated with lupus nephritis. Proc. Natl. Acad. Sci. USA.

[184] Leffler J., Martin M., Gullstrand B., Tyden H., Lood C., Truedsson L., Bengtsson A.A., Blom A.M. (2012). Neutrophil Extracellular Traps That Are Not Degraded in Systemic Lupus Erythematosus Activate Complement Exacerbating the Disease. J. Immunol..

[185] Dwivedi N., Upadhyay J., Neeli I., Khan S., Pattanaik D., Myers L., Kirou K.A., Hellmich B., Knuckley B., Thompson P.R. (2012). Felty’s syndrome autoantibodies bind to deiminated histones and neutrophil extracellular chromatin traps. Arthritis Rheum..

[186] Dwivedi N., Neeli I., Schall N., Wan H., Desiderio D.M., Csernok E., Thompson P.R., Dali H., Briand J.P., Muller S. (2014). Deimination of linker histones links neutrophil extracellular trap release with autoantibodies in systemic autoimmunity. FASEB J..

[187] Knight J.S., Zhao W., Luo W., Subramanian V., O’Dell A.A., Yalavarthi S., Hodgin J.B., Eitzman D.T., Thompson P.R., Kaplan M.J. (2013). Peptidylarginine deiminase inhibition is immunomodulatory and vasculoprotective in murine lupus. J. Clin. Investig..

[188] Knight J.S., Subramanian V., O’Dell A.A., Yalavarthi S., Zhao W., Smith C.K., Hodgin J.B., Thompson P.R., Kaplan M.J. (2015). Peptidylarginine deiminase inhibition disrupts NET formation and protects against kidney, skin and vascular disease in lupus-prone MRL/lpr mice. Ann. Rheum. Dis..

[189] Bolden J.E., Peart M.J., Johnstone R.W. (2006). Anticancer activities of histone deacetylase inhibitors. Nat. Rev. Drug Discov..

[190] Fiskus W., Ren Y., Mohapatra A., Bali P., Mandawat A., Rao R., Herger B., Yang Y., Atadja P., Wu J. (2007). Hydroxamic acid analogue histone deacetylase inhibitors attenuate estrogen receptor-α levels and transcriptional activity: A result of hyperacetylation and inhibition of chaperone function of heat shock protein 90. Clin. Cancer Res..

[191] Glozak M.A., Sengupta N., Zhang X., Seto E. (2005). Acetylation and deacetylation of non-histone proteins. Gene.

[192] Johnstone R.W. (2002). Histone-deacetylase inhibitors: Novel drugs for the treatment of cancer. Nat. Rev. Drug Discov..

[193] Park J.H., Jung Y., Kim T.Y., Kim S.G., Jong H.S., Lee J.W., Kim D.K., Lee J.S., Kim N.K., Kim T.Y. (2004). Class I histone deacetylase-selective novel synthetic inhibitors potently inhibit human tumor proliferation. Clin. Cancer Res..

[194] Huang B.H., Laban M., Leung C.H.-W., Lee L., Lee C.K., Salto-Tellez M., Raju G.C., Hooi S.C. (2005). Inhibition of histone deacetylase 2 increases apoptosis and p21Cip1/WAF1 expression, independent of histone deacetylase 1. Cell Death Differ..

[195] Swierczak A., Mouchemore K.A., Hamilton J.A., Anderson R.L. (2015). Neutrophils: Important contributors to tumor progression and metastasis. Cancer Metastasis Rev..

[196] Demers M., Wong S.L., Martinod K., Gallant M., Cabral J.E., Wang Y., Wagner D.D. (2016). Priming of neutrophils toward NETosis promotes tumor growth. Oncoimmunology.

[197] Pieterse E., Rother N., Garsen M., Hofstra J.M., Satchell S.C., Hoffmann M., Loeven M.A., Knaapen H.K., Van Der Heijden O.W.H., Berden J.H.M. (2017). Neutrophil Extracellular Traps Drive Endothelial-to-Mesenchymal Transition. Arterioscler. Thromb. Vasc. Biol..

[198] Cools-Lartigue J., Spicer J., McDonald B., Gowing S., Chow S., Giannias B., Bourdeau F., Kubes P., Ferri L. (2013). Neutrophil extracellular traps sequester circulating tumor cells and promote metastasis. J. Clin..

[199] Thålin C., Lundström S., Seignez C., Daleskog M., Lundström A., Henriksson P., Helleday T., Phillipson M., Wallén H., Demers M. (2018). Citrullinated histone H3 as a novel prognostic blood marker in patients with advanced cancer. PLoS ONE.

[200] Sen G.S., Mohanty S., Hossain D.M.S., Bhattacharyya S., Banerjee S., Chakraborty J., Saha S., Ray P., Bhattacharjee P., Mandal D. (2011). Curcumin enhances the efficacy of chemotherapy by tailoring p65NFκB-p300 cross-talk in favor of p53-p300 in breast cancer. J. Biol. Chem..

[201] Jiang L., Wang Y., Zhu D., Xue Z., Mao H. (2016). Alteration of histone H3 lysine 9 dimethylation in peripheral white blood cells of septic patients with trauma and cancer. Mol. Med. Rep..

[202] Simiele F., Recchiuti A., Patruno S., Plebani R., Pierdomenico A.M., Codagnone M., Romano M. (2016). Epigenetic regulation of the formyl peptide receptor 2 gene. Biochim. Biophys. Acta Gene Regul. Mech..

[203] Wang B., Wang X.B., Chen L.Y., Huang L., Dong R.Z. (2013). Belinostat-induced apoptosis and growth inhibition in pancreatic cancer cells involve activation of TAK1-AMPK signaling axis. Biochem. Biophys. Res. Commun..

[204] Lin S.F., Lin J.D., Chou T.C., Huang Y.Y., Wong R.J. (2013). Utility of a Histone Deacetylase Inhibitor (PXD101) for Thyroid Cancer Treatment. PLoS ONE.

